# Exploring Proteomes of Robust Yarrowia lipolytica Isolates Cultivated in Biomass Hydrolysate Reveals Key Processes Impacting Mixed Sugar Utilization, Lipid Accumulation, and Degradation

**DOI:** 10.1128/mSystems.00443-21

**Published:** 2021-08-03

**Authors:** Caleb Walker, Bruce Dien, Richard J. Giannone, Patricia Slininger, Stephanie R. Thompson, Cong T. Trinh

**Affiliations:** a Department of Chemical and Biomolecular Engineering, University of Tennessee, Tennessee, USA; b Bioenergy Research Unit, The National Center for Agricultural Utilization Research, USDA-ARS, Peoria, Illinois, USA; c Biosciences Division, Oak Ridge National Laboratory, Oak Ridge, Tennessee, USA; Northwestern University

**Keywords:** bioreactor characterization, proteomic analysis, xylose metabolism, xylose transporters, lipid accumulation, lipid degradation, lipid regulators, proteome, proteomic analysis, robustness, *Yarrowia lipolytica*

## Abstract

Yarrowia lipolytica is an oleaginous yeast exhibiting robust phenotypes beneficial for industrial biotechnology. The phenotypic diversity found within the undomesticated Y. lipolytica clade from various origins illuminates desirable phenotypic traits not found in the conventional laboratory strain CBS7504 (or W29), which include xylose utilization, lipid accumulation, and growth on undetoxified biomass hydrolysates. Currently, the related phenotypes of lipid accumulation and degradation when metabolizing nonpreferred sugars (e.g., xylose) associated with biomass hydrolysates are poorly understood, making it difficult to control and engineer in Y. lipolytica. To fill this knowledge gap, we analyzed the genetic diversity of five undomesticated Y. lipolytica strains and identified singleton genes and genes exclusively shared by strains exhibiting desirable phenotypes. Strain characterizations from controlled bioreactor cultures revealed that the undomesticated strain YB420 used xylose to support cell growth and maintained high lipid levels, while the conventional strain CBS7504 degraded cell biomass and lipids when xylose was the sole remaining carbon source. From proteomic analysis, we identified carbohydrate transporters, xylose metabolic enzymes, and pentose phosphate pathway proteins stimulated during the xylose uptake stage for both strains. Furthermore, we distinguished proteins involved in lipid metabolism (e.g., lipase, NADPH generation, lipid regulators, and β-oxidation) activated by YB420 (lipid maintenance phenotype) or CBS7504 (lipid degradation phenotype) when xylose was the sole remaining carbon source. Overall, the results relate genetic diversity of undomesticated Y. lipolytica strains to complex phenotypes of superior growth, sugar utilization, lipid accumulation, and degradation in biomass hydrolysates.

**IMPORTANCE** Yarrowia lipolytica is an important industrial oleaginous yeast due to its robust phenotypes for effective conversion of inhibitory lignocellulosic biomass hydrolysates into neutral lipids. While lipid accumulation has been well characterized in this organism, its interconnected lipid degradation phenotype is poorly understood during fermentation of biomass hydrolysates. Our investigation into the genetic diversity of undomesticated Y. lipolytica strains, coupled with detailed strain characterization and proteomic analysis, revealed metabolic processes and regulatory elements conferring desirable phenotypes for growth, sugar utilization, and lipid accumulation in undetoxified biomass hydrolysates by these natural variants. This study provides a better understanding of the robust metabolism of Y. lipolytica and suggests potential metabolic engineering strategies to enhance its performance.

## INTRODUCTION

Yarrowia lipolytica is an important oleaginous yeast for industrial biotechnology. Wild-type strains can accumulate a remarkable 40% of cell weight in neutral lipids from lignocellulosic biomass or agricultural wastes (1). These microbial lipids are a promising alternative to petroleum and animal oils for the sustainable production of advanced fuels and oleochemicals. In addition, Y. lipolytica is exceptionally robust to chemical inhibitors and stressful environments, which are critical biocatalyst properties to achieve sustainable production of chemicals from low-cost biomass feedstocks. The yeast can tolerate broad pH ranges (2), high salt concentrations (3), and organic solvents (e.g., ionic liquids) (4, 5); in fact, most Y. lipolytica isolates exhibit robust growth in up to 60% (vol/vol) undetoxified dilute acid-pretreated switchgrass hydrolysates that are normally inhibitory to microbes (6). Thus, a better understanding of the mechanisms that underpin Y. lipolytica’ s natural robustness would not only enable development of niche strains for novel biocatalysis but would also provide fundamental knowledge that may be applied to other industrially relevant organisms.

Recently, significant research has focused on manipulating the metabolism of the conventional PO1 series laboratory strain CBS7504 (W29), isolated from a Paris sewer and well domesticated in the laboratory (7), for enhanced lipid production and utilization of pentose (e.g., xylose) and hexose (e.g., glucose) sugars in inhibitory lignocellulosic biomass hydrolysates. To increase lipid production in Y. lipolytica, numerous metabolic engineering strategies have been implemented that successfully redirected carbon flux to lipid metabolism (8), including overexpression of lipid biosynthesis enzymes (9) and/or disruption of the competitive β-oxidation pathway (10), as well as altered expression of regulators (e.g., SNF1) of lipid accumulation (11). The most lipogenic Y. lipolytica strain reported to date achieved 90% lipid content by simultaneous restoration of leucine and uracil biosynthesis, overexpression of diacylglycerol transferase (DGA1), deletion of peroxisome biogenesis enzyme peroxin-10 (PEX10), deletion of multifunctional β-oxidation enzyme (MFE1), and optimization of culture conditions (12).

To maximize lipogenesis from biomass hydrolysates requires efficient utilization of both hexose and pentose sugars. Y. lipolytica does not efficiently use xylose as a sole carbon source, albeit it processes genes for the complete xylose catabolic pathway (13). Activation of this cryptic pathway has been accomplished through adaptive evolutionary approaches resulting in improved xylose utilization (14). Furthermore, overexpression of endogenous xylose catabolic genes (15–17) and heterologous expression of xylose reductase and xylitol dehydrogenase from the yeast Scheffersomyces stipitis (17, 18) have successfully increased xylose consumption rates. Several transporters have also been identified in Y. lipolytica that show increased expression levels during xylose assimilation, and combinatorial overexpression of the endogenous xylitol dehydrogenase with several of these transporters has also resulted in improved growth on xylose (19). While the production phenotypes are well characterized, fundamental understanding of complex phenotypes responsible for superior growth, sugar utilization, and lipid accumulation—or degradation—during fermentation of biomass hydrolysates is still lacking.

Complementary to these engineering efforts, recent investigation into the genetic diversity of undomesticated Y. lipolytica strains revealed emergent robust phenotypes not present in the conventional strain CBS7504. Characterization of 57 undomesticated Y. lipolytica isolates on inhibitory undetoxified biomass hydrolysates revealed select strains with enhanced growth, lipid production, and pentose-sugar assimilation relative to CBS7504 (6). In this study, Y. lipolytica’ s natural genetic diversity is further explored using a combination of detailed strain characterization and proteomic analysis. Coupled together, these analyses uncover the underlying mechanisms behind these poorly understood complex phenotypes during fermentation of biomass hydrolysates. The results presented here will aid engineering efforts to better control lipid accumulation or degradation phenotypes for optimally producing advanced biofuels and/or oleochemicals from biomass hydrolysates.

## RESULTS

### Comparative genomics reveals unique genotypes of undomesticated *Yarrowia* strains.

**FIG 1:**
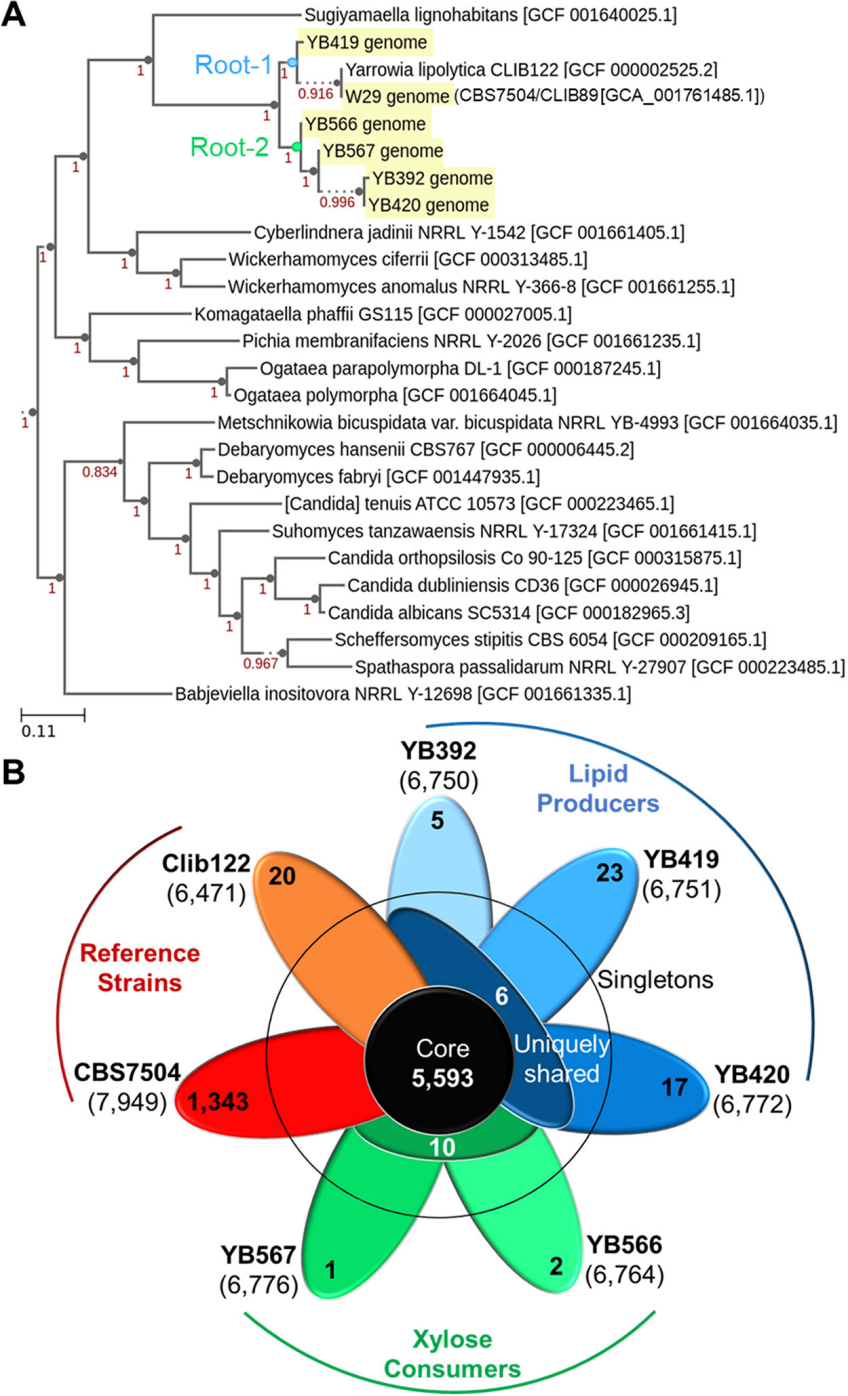
(A) Phylogenetic tree of Y. lipolytica isolates with 20 closest neighbor species. (B) Pangenome of Y. lipolytica reference strains CLIB122122 and CBS7504 and undomesticated strains YB392, YB419, YB420, YB566, and YB567. Boldface, strain names; parentheses, total genes; outer petals, singleton genes, middle petals, uniquely shared genes between lipid producers (blue) and xylose consumers (green); middle circle, core genes.

**Phylogenetic tree of** **Y. lipolytica** **isolates shows close similarity between genomes.** Phylogenetic species analysis distinguished the first evolutionary split dividing the *Yarrowia* clade into two ancestral roots (Fig. 1A). The first root contained the undomesticated YB419 and the conventional strains CBS7504 and CLIB122 (a species crossed between CBS7504 and CBS6124-2 [7]). The second root contained the remaining four nonconventional isolates, depicting YB392 and YB420 as the most divergent from YB567, followed by YB566. This result was surprising, since YB392, YB419, and YB420 were all isolated from corn milling plants within Illinois (20). Interestingly, the closest related species to the *Yarrowia* clade is Sugiyamaella lignohabitans, an efficient pentose-utilizing and facultative anaerobic yeast (21).

**Undomesticated** **Y. lipolytica** **isolates contain unique genes not found in conventional strains.** The undomesticated strains were characterized for unique genes that may contribute to their distinctive phenotypes. A singleton gene signifies a gene appearing exclusively in one of the genomes within the pangenome (i.e., conventional strains CBS7504 and CLIB122; undomesticated strains YB392, YB419, YB420, YB566, and YB567). Of the undomesticated Y. lipolytica strains, YB419 contained the most singletons (23 genes) followed by YB420 (17 genes), while the remaining 3 isolates contained 5 or fewer singletons (Fig. 1B, Table S1 in the supplemental material). Two of the undomesticated strains, YB566 and YB567, exhibiting better xylose assimilation from switchgrass hydrolysates (SGH) (6), exclusively share 10 genes not found in the other three strains. (Fig. 1B, Table S2). Likewise, six genes are exclusively shared among the undomesticated strains YB392, YB419, and YB420, all exhibiting better lipid accumulation (Fig. 1B, Table S2) (6).

**FIG 2:**
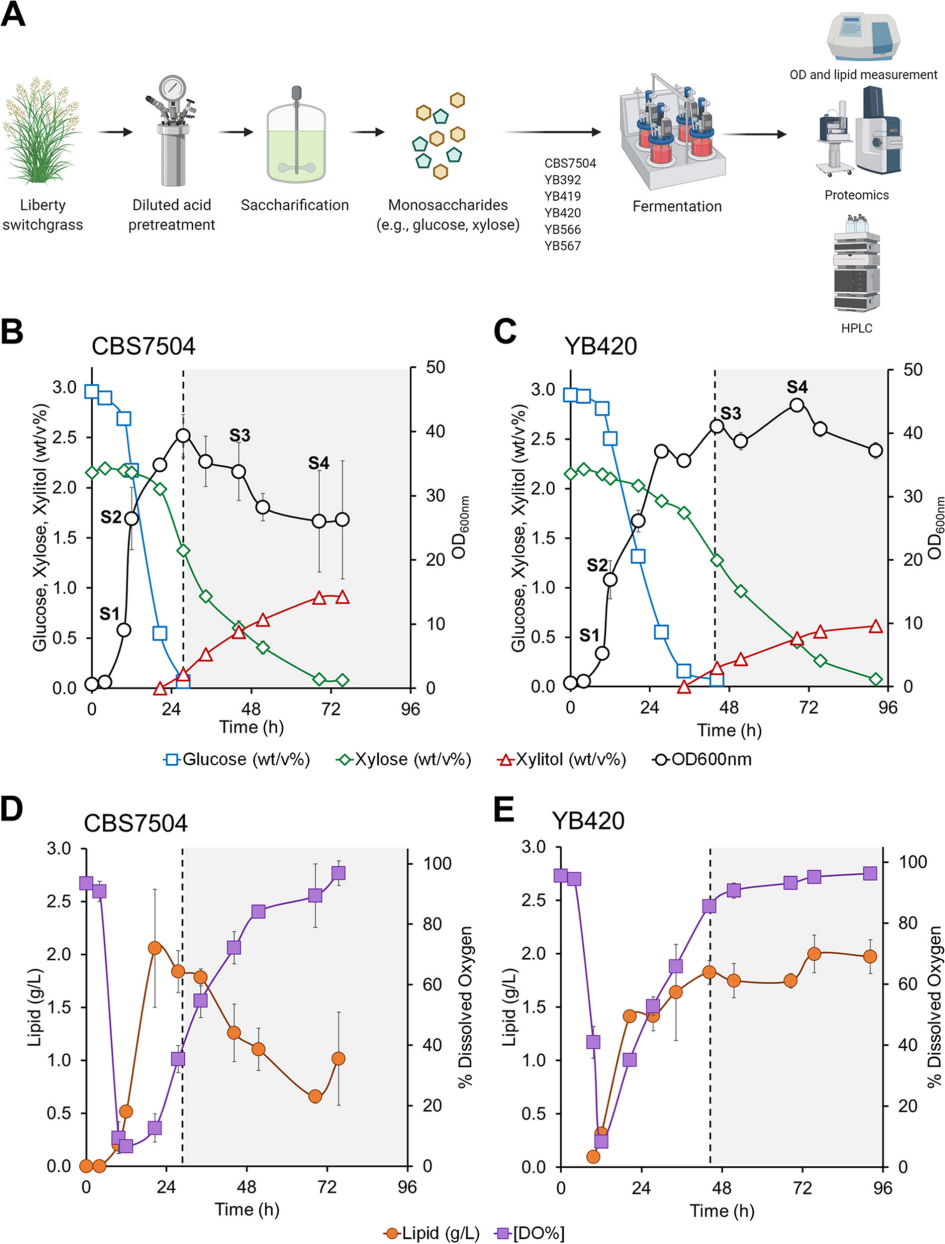
(A) Workflow of bioreactor characterization of Y. lipolytica isolates CBS7504 (B and D) and YB420 (C and E) in 50% switchgrass hydrolysate (SGH). Dotted lines, time of glucose depletion where xylose is the sole remaining carbon source. Error bars represent the standard deviation between biological triplicates (*n* = 3).

**Y. lipolytica** **strains of different origins thrive in undetoxified biomass hydrolysates and exhibit distinct phenotypes.** To better understand how the genetic diversity influences the robust phenotypes with respect to cell growth, mixed sugar co-utilization, and lipid accumulation, we characterized CBS7504 (Fig. S1), YB392 (Fig. S2), YB419 (Fig. S3), YB420 (Fig. S4), YB566 (Fig. S5), and YB567 (Fig. S6) in 50% (vol/vol) undetoxified biomass hydrolysates. Unlike the previous studies using deep-well microplates and flasks (6), we characterized these strains in computer-controlled bioreactors (Fig. 2A). In general, all strains grew well in undetoxified biomass hydrolysates. However, there were distinct phenotypes among strains associated with cell growth, mixed sugar co-utilization, and lipid accumulation. Here, two representative Y. lipolytica strains, CBS7504 and YB420, were selected for in-depth analysis due to their distinctive differences in xylose and lipid metabolism and membership in two different ancestral roots.

The conventional Y. lipolytica strain CBS7504 grew well in undetoxified biomass hydrolysates, achieving maximum cell mass (39.3 ± 3.3 at optical density of 600 nm [OD_600_], where an OD_600_ of 1= 0.34 g/liter) within 28 h of fermentation from the co-utilization of 2.90 ± 0.05 (% wt/vol) glucose and 0.80 ± 0.03 (% wt/vol) xylose (Fig. 2B). Lipids accumulated during growth, reaching a maximum of 2.10 ± 0.60 g/liter at 21 h into the fermentation (Fig. 2D). Upon glucose exhaustion, cell mass and accumulated lipid levels steadily declined for the remaining 48 h of fermentation, despite the continued consumption of xylose. At 72 h of fermentation, CBS7504 consumed a total of 2.10 ± 0.02 (% wt/vol) xylose and produced 0.91 ± 0.01 (% wt/vol) xylitol (yielding 0.44 ± 0.00 xylitol/xylose). The undomesticated Y. lipolytica strain YB420 also grew robustly in undetoxified biomass hydrolysates, but showed contrasting phenotypes with CBS7504. Over 45 h of fermentation, YB420 showed less co-utilization of glucose (2.80 ± 0.03 [% wt/vol]) and xylose (0.40 ± 0.03 [% wt/vol]) and less lipid production (1.6 ± 0.5 g/liter) than CBS7504 (Fig. 2C and E). However, upon glucose depletion, YB420 maintained cell mass and lipids while consuming a total of 2.1 ± 0.01 (% wt/vol) xylose and producing 0.61 ± 0.04 (% wt/vol) xylitol (yielding 0.30 ± 0.006 xylitol/xylose). The differences observed in xylose utilization to support maintenance of lipids and cell growth prompted a systems-level comparison between the two strains CBS7504 and YB420.

### Proteomic analysis reveals key processes impacting sugar utilization and lipid degradation.

**FIG 3:**
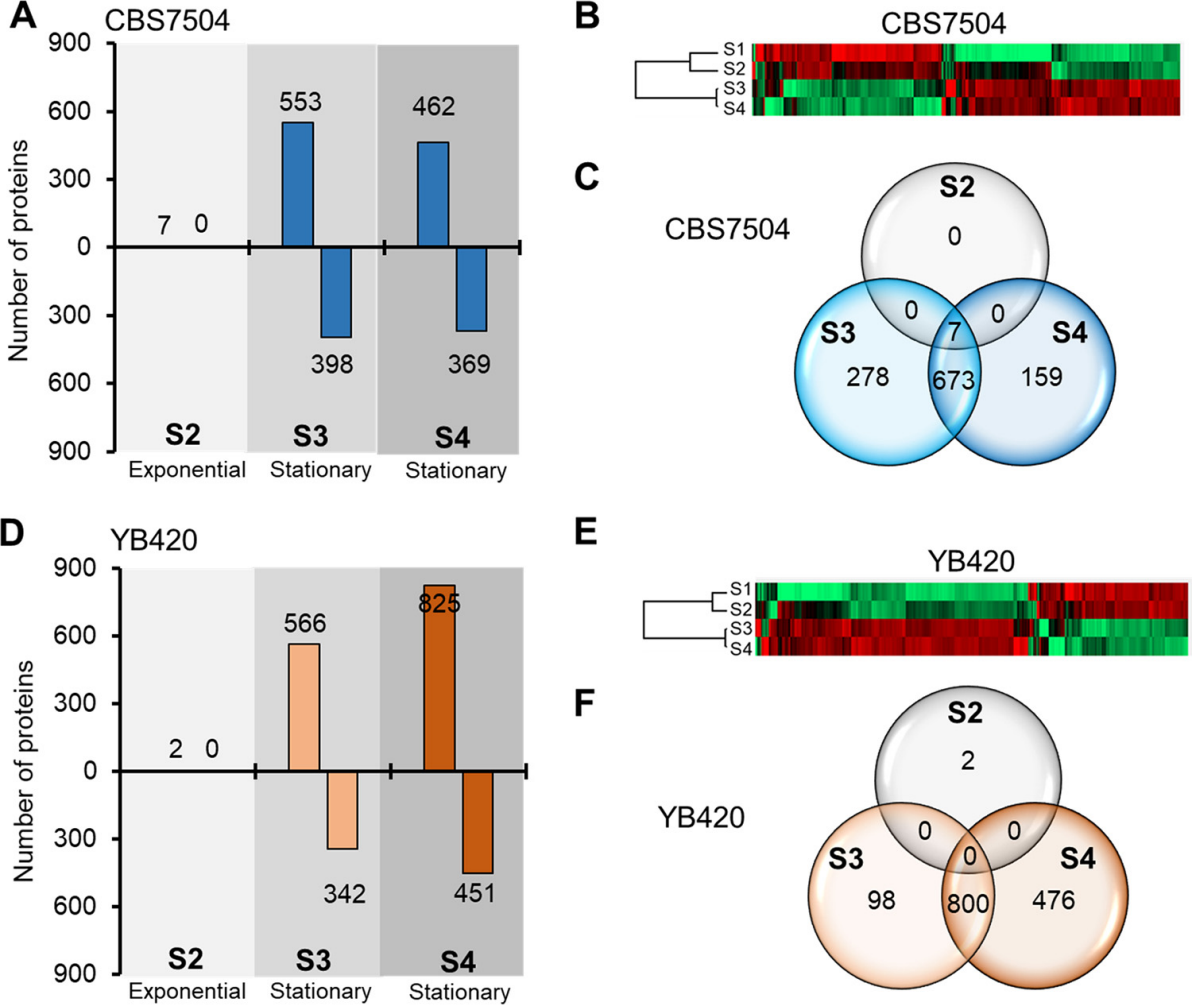
Proteomic analysis of Y. lipolytica CBS7504 (A to C) and YB420 (D to F) strains. (A and D) Number of proteins with significant abundance changes relative to S1. (B and E) Heatmaps of significant proteins with changed abundance relative to S1. (C and F) Venn diagrams illustrating the number of proteins with significant abundance changes between samples.

**Proteome alterations in growth stages.** Proteomic samples were collected during the exponential growth phase when glucose was assimilated (S1 and S2) and stationary phase when xylose was assimilated (S3 and S4) for both strains in biomass hydrolysate cultures. Differences in protein abundances were compared for S2, S3, and S4 against S1 for each strain (Fig. 3A, B, D, and E). As expected, there were only minor proteome differences between glucose-assimilation-phase samples (S1 and S2) (Fig. 3A and D). However, Y. lipolytica strains dramatically altered their proteomes during stationary-phase samples (samples S3 and S4), when xylose was assimilated, and lipid levels were maintained by YB420 but degraded by CBS7504 (Fig. 3B and E). For CBS7504, 673 protein abundances changed throughout the stationary phase (samples S3 and S4) (Fig. 3C), while 800 protein abundances were changed in YB420 (Fig. 3F). Since the largest proteome differences were found between exponential (S1 and S2) and stationary (S3 and S4) phase samples, we chose to characterize xylose assimilation and lipid degradation phenotypes using proteins with significant changes in abundance at S3 and/or S4 relative to S1.

**FIG 4:**
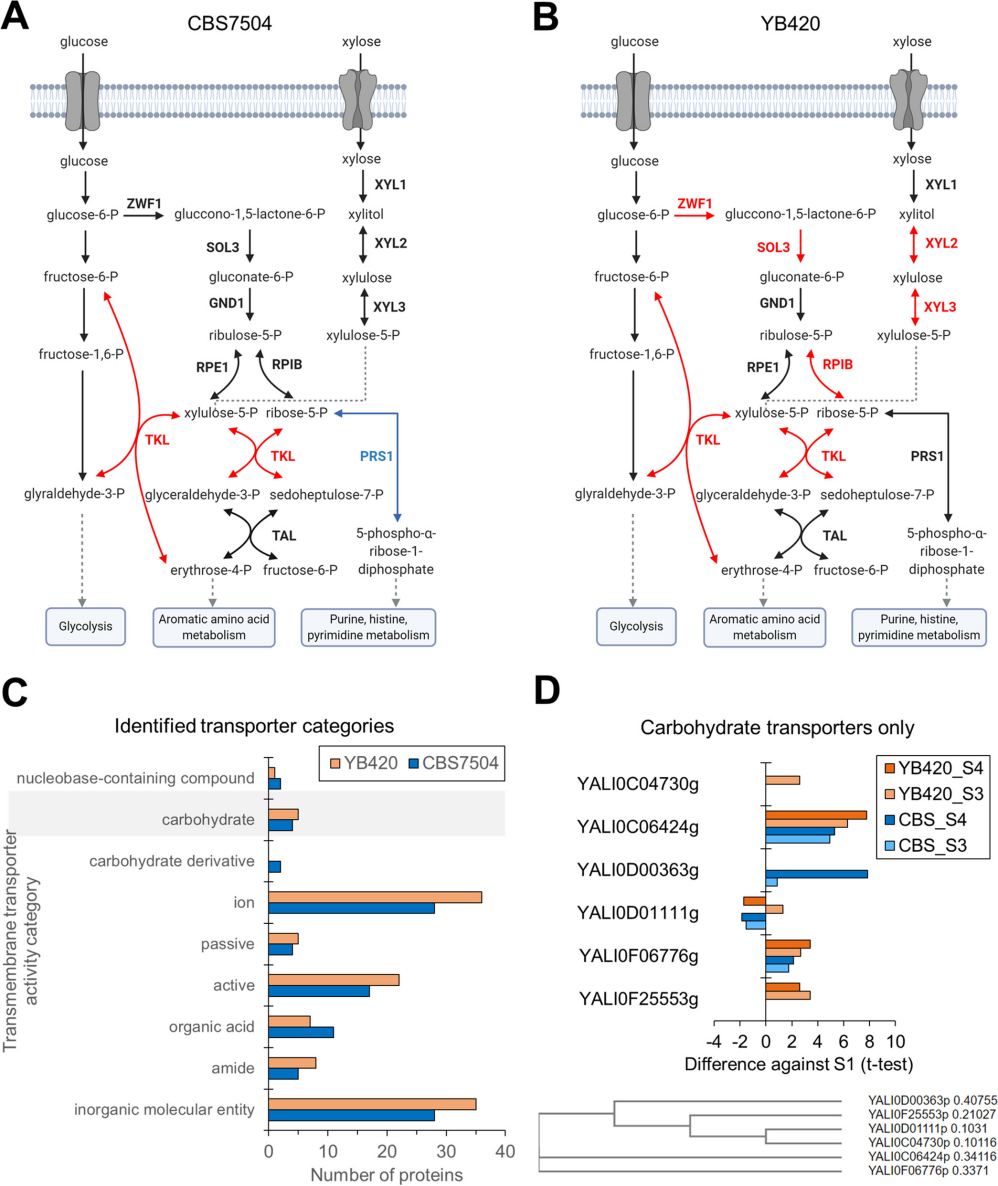
Pentose phosphate pathway proteome of CBS7504 (A) and YB420 (B). Increased protein abundance during xylose assimilation, red; decreased protein abundance during xylose assimilation, blue; pathways, rectangles; proteins, boldface text; metabolites, plain text. (C and D) All transporters (C) and carbohydrate transporters (D) with significant protein abundance changes in the xylose assimilation phase. CBS7504, blue; YB420, orange. Abbreviations: TKL (transketolase, YALI0D02277g); PRS1 (ribose-phosphate pyrophosphokinase, YALI0B13552g); Xyl1 (xylose reductase, YALI0D07634g); Xyl2 (xylitol dehydrogenase, YALI0E12463g); Xyl3 (xylulokinase, YALI0F10923g); ZWF1 (NADP^+^-dependent glucose-6-phosphate dehydrogenase, YALI0E22649g); SOL3 (6-phosphogluconolactonase, YALI0E11671g); RPIB (ribose 5-phosphate isomerase, YALI0F01628g); TAL (transaldolase, YALI0F15587g); RPE1 (ribulose-phosphate 3-epimerase, YALI0C11880g); GND1 (6-phosphogluconate dehydrogenase, YALI0B15598g).

**Proteome alterations in xylose assimilation.** CBS7504 consumed xylose slightly faster than YB420 but converted more of it into xylitol (0.44 ± 0.002% wt/wt of xylose) rather than maintaining cell mass or lipid content (Fig. 2B and C). This led us to investigate protein abundances in the pentose phosphate pathway (PPP), where xylose is introduced into central metabolism. Despite the quick assimilation of xylose, strain CBS7504 during xylose assimilation (S3 and S4) only upregulated the protein abundance of transketolase (TKL, YALI0D02277g) in the PPP (Fig. 4A, Table S3). Interestingly, CBS7504 downregulated the protein abundance of ribose-phosphate pyrophosphokinase (PRS1, YALI0B13552g), which converts ribose-5-phosphate into 5-phosphoribosyl 1-pyrophosphate (PRPP) to feed downstream biosynthetic pathways associated with cell growth (i.e., histidine, pyrimidine, and purine metabolism). This downregulation of PRS1 correlates with the decreased cell mass, increased xylitol production, and lipid degradation phenotypes of CBS7504. Meanwhile, during xylose assimilation (S3 and S4), YB420 produced less xylitol (0.30 ± 0.006% wt/wt of xylose) and maintained cell mass and lipid content (Fig. 2C). Regarding the PPP, six proteins were upregulated and none downregulated in YB420 during the stationary phase (Fig. 4B, Table S3). Not surprisingly, these upregulated proteins include xylitol dehydrogenase (Xyl2, YALI0E12463g) and xylulokinase (Xyl3, YALI0F10923g), which together convert xylitol into xylulose-5P. Notably, YB420 also upregulated 2 proteins annotated (in panther database) for ribulo and xylulose kinase (YALI0D15114g) and ribulokinase (YALI0E13321g) activities. Additionally, YB420 increased the protein abundance of D-arabinitol 2-dehydrogenase (ADH, YALI0F02211g).

**Transporters.** In total, 87 transporters were identified with statistically significant abundance changes when comparing stationary phase (S3 and/or S4) to exponential growth phase (S1) (Fig. 4C, Table S3). While most are annotated for ion and inorganic molecular entity transmembrane transport activities, we focused on the 28 active transmembrane transporters to identify those with altered protein abundances during xylose assimilation. Specifically, six of these transporters are annotated with carbohydrate transmembrane transporter activity and have been studied for xylose assimilation in Y. lipolytica (Fig. 4D) (14). YALI0C06424g is a carbohydrate symporter with the largest increase in abundance for both strains. This protein is similar to the Snf3p and Rgt2p proteins of Saccharomyces cerevisiae, which are involved in glucose sensing and signaling, as well as fructose and mannose transport (22). Both CBS7504 and YB420 strains also increased the abundance of YALI0F06776g, an observation in agreement with previous transcriptomics data measuring Y. lipolytica’s growth response to xylose as the sole carbon source (14). While these two transporters exhibited similar upregulation patterns during growth on xylose across both strains, albeit with varied magnitudes, other transporters were more strain specific, such as YALI0D00363g, YALI0F25553g, and YALI0C04730g. YALI0D00363g was strongly upregulated in S4 for CBS7504, but not in YB420, while YB420 increased the protein abundance of YALI0F25553g at both S3 and S4 and YALI0C04730g at S3, which was not seen in CBS7504. Interestingly, individual overexpression of any of these three carbohydrate symporters (YALI0D00363g, YALI0F25553g, or YALI0C04730g) supported growth of Y. lipolytica PO1f on plates containing xylose as the sole carbon source (19). Lastly, YALI0D01111g was downregulated in CBS7504 during xylose assimilation at S3 and S4 and at S4 in YB420.

**FIG 5:**
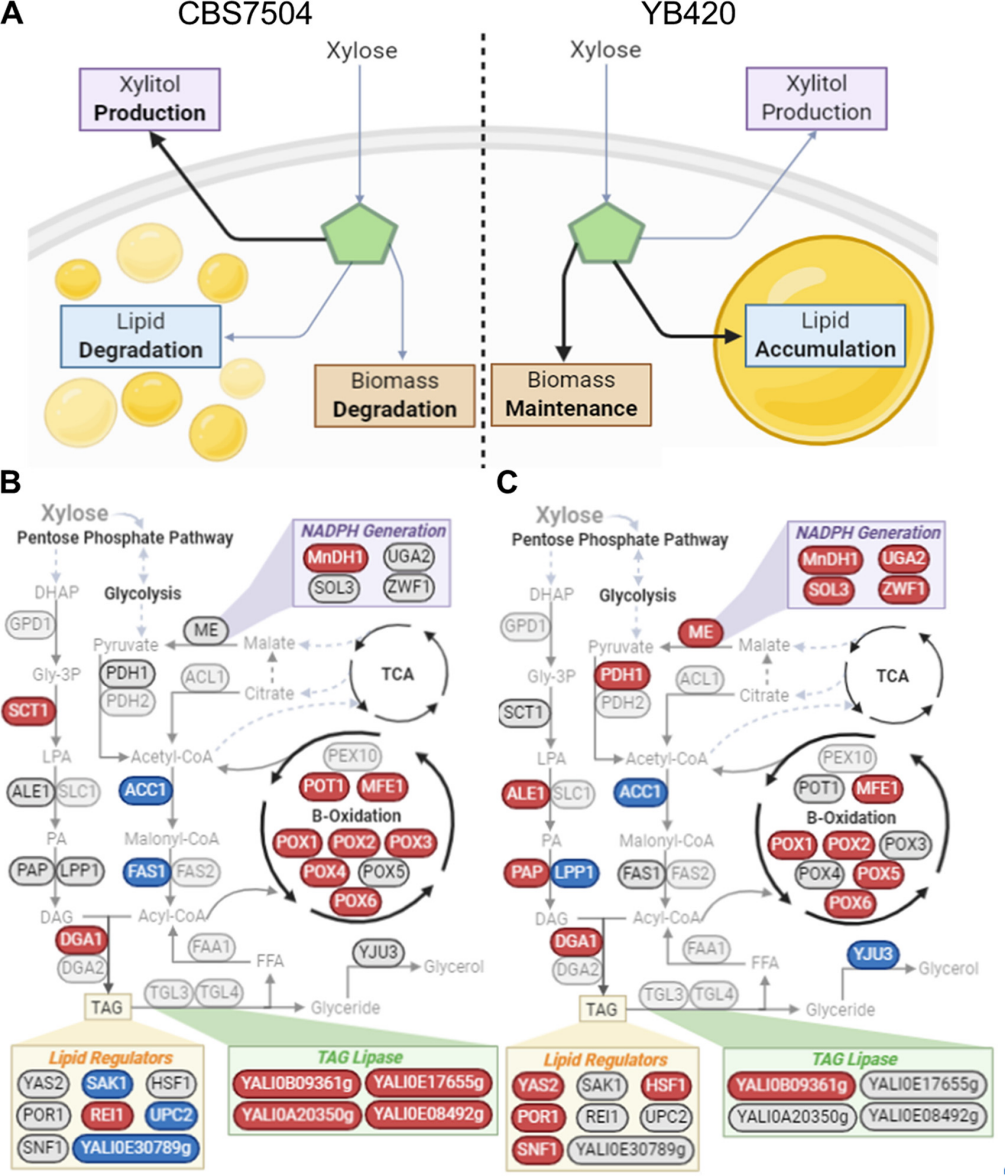
(A) Schematic of growth characterization phenotypes for CBS7504 and YB420 when xylose was the sole remaining carbon source. (B and C) Proteomic analysis of lipid metabolism showing increased (red) and decreased (blue) protein abundance when xylose was the sole remaining carbon source for CBS7504 (B) and YB420 (C). Proteins, circles; metabolites, plain text; pathways, boldface text. Abbreviations: TAG (triacylglycerol); DHAP (dihydroxyacetone phosphate); Gly-3P (glycerol-3-phosphate); LPA (lysophosphatidic acid); PA, phosphatidic acid; DAG, diacylglycerol; PL, phospholipid; TAG, triacylglycerol; FFA, free fatty acid; TCA, tricarboxylic acid cycle; GPD1 (glycerol-3-phosphate dehydrogenase, YALI0B02948g); SCT1 (bifunctional glycerol-3-phosphate/glycerone-phosphate *O*-acyltransferase, YALI0C00209g); ALE1 (lysophosphatidate acyltransferase, YALI0F19514g); SLC1 (1-acyl-sn-glycerol-3-phosphate acyltransferase, YALI0E18964g); PAP (phosphatidic acid phosphohydrolase, YALI0D27016g); LPP1 (diacylglycerol diphosphate phosphatase/phosphatidate phosphatase, YALI0B14531g); DGA1 (acyl-CoA dependent diacylglycerol acyltransferase I, YALI0E32769g); DGA2 (acyl-CoA dependent diacylglycerol acyltransferase II, YALI0D07986g); TGL3 (triacylglycerol lipases 3, YALI0D17534g); TGL4 (triacylglycerol lipase 4, YALI0F10010g); YJU3 (monoglycerol lipase, YALI0C14520g); FAA1 (long-chain acyl-CoA synthetase, YALI0D17864g); FAS1 (fatty acid synthase 1, YALI0B15059g); FAS2 (fatty acid synthase 2; YALI0B19382g); ACC1 (acetyl-CoA carboxylase, YALI0C11407g); PDH1, PDH2; ACL1 (ATP-citrate lyase, YALI0E34793g); ME (malic enzyme, YALI0E18634g); MnDH1 (Mannitol dehydrogenase, YALI0B16192g); UGA2 (Succinate semialdehyde dehydrogenase, YALI0F26191g); SOL3 (6-Phosphogluconolactonase, YALI0E11671g); ZWF1 (NADP+-dependent glucose-6-phosphate dehydrogenase, YALI0E22649g); POT1 (3-oxyacyl-thiolase, YALI0E18568g); MFE1 (multifunctional β-oxidation protein, YALI0E15378g); PEX10 (peroxin-10, YALI0C01023g); POX1 (acyl-coenzyme A oxidase 1, YALI0E32835g); POX2 (acyl-coenzyme A oxidase 2, YALI0F10857g); POX3 (acyl-coenzyme A oxidase 3, YALI0D24750g); POX4 (acyl-coenzyme A oxidase 4, YALI0E27654g); POX5 (acyl-coenzyme A oxidase 5, YALI0C23859g); POX6 (acyl-coenzyme A oxidase 6, YALI0E06567g); YAS2 (HLH transcription factor, YALI0E32417g); POR1 (YALI0D12628g); SNF1 (YALI0D02101g); SAK1 (YALI0D08822g); REI1 (YALI0B08734g); HSF1 (YALI0E13948g); UPC2 (YALI0B15818g);.

**Proteome alterations in lipid metabolism.** CBS7504 demonstrated lipid degradation while YB420 maintained lipid content during the stationary/xylose assimilation growth stage samples (S3 and/or S4) (Fig. 2D and E, Fig. 5A). To understand the cause of lipid degradation versus maintenance, we compared proteins involved in fatty acid degradation and triacylglycerol (TAG) metabolism. In the TAG synthesis pathway, CBS7504 increased the abundance of both bifunctional glycerol-3-phosphate/glycerone-phosphate *O*-acyltransferase (SCT1, YALI0C00209g), converting glycerol-3-phosphate (gly-3P) into lysophosphatidic acid (LPA), and acyl-CoA dependent diacylglycerol acyltransferase I (DGA1, YALI0E32769g), which converts diacylglycerol (DAG) into TAG (Fig. 5B, Table S3). Likewise, YB420 increased the abundance DGA1, but also lysophosphatidate acyltransferase (ALE1, YALI0F19514g) and phosphatidic acid phosphohydrolase (PAP, YALI0D27016g), which together convert LPA into TAG (Fig. 5C, Table S3). However, YB420 downregulated the abundance of diacylglycerol diphosphate phosphatase/phosphatidate phosphatase (LPP1, YALI0B14531g), which has the same metabolic function as PAP (KEGG E.C.3.1.3.4).

Considering the β-oxidation pathway, both strains showed increased protein abundance of acyl-coenzyme A oxidases 1 (POX1, YALI0E32835g), 2 (POX2, YALI0F10857g), and 6 (POX6, YALI0E06567g) and multifunctional β-oxidation protein (MFE1, YALI0E15378g) (Fig. 5A and B). However, CBS7504 also showed increased abundance of POX3 (YALI0D24750g), POX4 (YALI0E27654g), and 3-oxyacyl-thiolase (POT1, YALI0E18568g), all involved in the breakdown of TAGs into free fatty acids (FFA). Both strains decreased the abundance of acetyl-CoA carboxylase (ACC1, YALI0C11407g), but CBS7504 also decreased abundance of fatty acid synthase subunit 1 (FAS1, YALI0B15059g). Interestingly, YB420 increased the abundance of malic enzyme (ME, YALI0E18634g), which generates NADPH pools that are required for the FAS complex in oleaginous organisms (23), but shows little to no involvement in lipid production in Y. lipolytica (24–26). The fact that the lipid level of YB420 remained unchanged suggests that the rates of lipid synthesis and degradation were similar once glucose was consumed.

**Lipase.** In total, 10 lipases were identified with statistically significant abundance changes during the stationary phase (S3 and/or S4) relative to exponential growth phase (S1) (Fig. 5B and C; Table S3). While none include the well-studied triacylglycerol lipase 3 (TGL3, YALI0D17534g) or TGL4 (YALI0F10010g), four of the identified lipases are annotated for TGL activity (PTHR23025). Interestingly, CBS7504 increased the protein abundance of all four TGLs in stationary phase, while YB420 only increased the protein abundance of one.

**NADPH generation.** We identified five differentially abundant proteins involved in the generation of NADPH, the reducing equivalent required to sustain fatty acid synthesis (Fig. 5B and C; Table S3). While both YB420 and CBS7504 strains increased the protein abundance of sorbitol dehydrogenase (MnDH1, YALI0B16192g) during stationary phase, only YB420 increased abundances of the other 4 proteins, including malic enzyme (ME, YALI0E18634g), succinate semialdehyde dehydrogenase (UGA2, YALI0F26191g), 6-phosphogluconolactonase (SOL3, YALI0E11671g), and NADP-dependent glucose-6-phosphate dehydrogenase (ZWF1, YALI0E22649g).

**Regulators of lipid synthesis.** Nitrogen limitation (i.e., high carbon to nitrogen ratio) is a common strategy to increase lipid synthesis from glucose (27), and numerous studies have reported regulators of lipid accumulation and genes affected by nitrogen limitation. Our analysis identified eight of these regulators with statistically significant abundance changes during the stationary phase (S3 and/or S4) relative to exponential growth phase (S1), all of which have been previously reported to influence lipid accumulation (11, 28, 29). Of these, YB420 increased the protein abundance of HLH transcription factor YAS2 (YALI0E32417g), a subunit of the SWI/SNF chromatin remodeling complex POR1 (YALI0D12628g), AMP-activated serine/threonine protein kinase SNF1 (YALI0D02101g), and heat shock transcription factor HSF1 (YALI0E13948g) (Fig. 5C, Table S3). Meanwhile, CBS7504 increased the protein abundance of cytoplasmic pre-60S factor REI1 (YALI0B08734g) and decreased the protein abundance of a zinc finger protein (YALI0E30789g), sterol regulatory element binding protein UPC2 (YALI0B15818g), and SNF1-activating kinase 1 SAK1 (YALI0D08822g) (Fig. 5B, Table S3).

**FIG 6:**
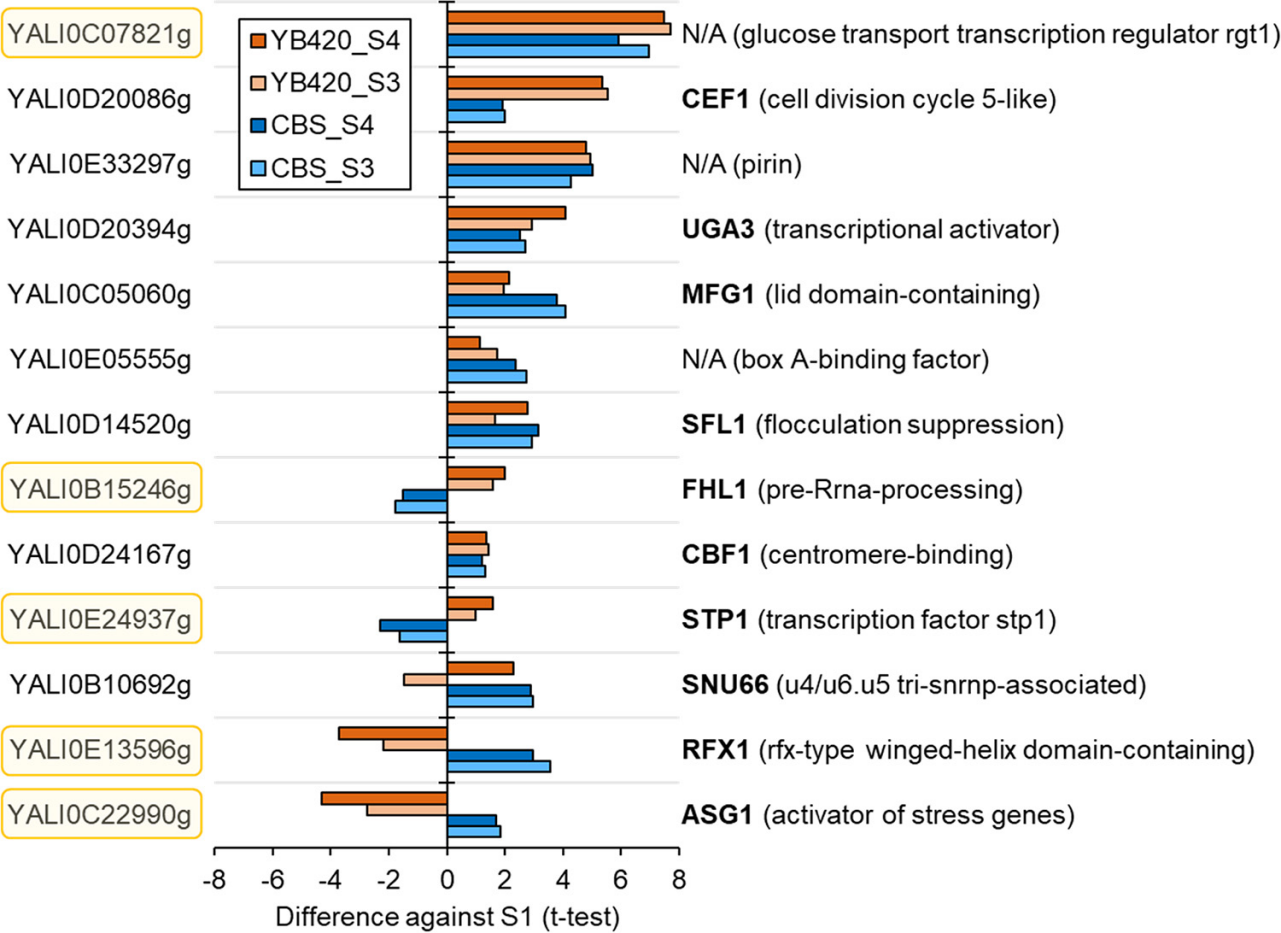
Regulator proteins with significant protein abundance changes in the xylose assimilation phase. CBS7504, blue; YB420, orange.

**Proteome alterations in regulatory elements**. In total, 46 gene-specific regulator proteins (from panther database) were identified with statistically significant abundance changes during the stationary phase (S3 and/or S4) relative to exponential growth phase (S1) (Fig. 6, Table S3). Thirteen of these regulatory proteins had significant changes in both CBS7504 and YB420 (Fig. 6). The protein with the largest increase in abundance, YALI0C07821g, is annotated as glucose transport transcription regulator RGT1-related (PTHR31668). Interestingly, four of these regulatory proteins have distinct, strain-specific abundance patterns: RFX1, ASG1, STP1, and FHL1. RFX1, regulatory factor X in S. cerevisiae, is a major transcriptional repressor of DNA-damage-regulated genes (30). ASG1, an activator of stress-related genes, activates genes in β-oxidation, glucogenesis, glyoxylate cycle, triacylglycerol breakdown, and peroxisomal transport, and helps assimilate fatty acids in S. cerevisiae (31). STP1, involved in species-specific tRNA processing, activates transcription of amino acid permease genes and is directly involved in pre-tRNA splicing in S. cerevisiae (32, 33). Finally, FHL1 (fork-head like) in S. cerevisiae functions as a transcription regulator of ribosomal protein transcription (34).

## DISCUSSION

Lipid accumulation and lipid degradation patterns from cultures using nonpreferred sugars prevalent in biomass hydrolysates (i.e., xylose) are complex phenotypes making them difficult to control and engineer in Y. lipolytica (15, 35, 36). By comparing proteomes of natively robust undomesticated Y. lipolytica strain YB420 with the conventional strain CBS7504, we identified key proteins supporting cell growth and lipid accumulation with xylose as the sole remaining carbon source.

Once all the glucose was consumed, YB420 continued to accumulate lipids and sustained cell mass from xylose, while CBS7504 degraded lipids, decreased cell mass, and produced more xylitol (Fig. 2). This more efficient use of xylose demonstrated by YB420 is supported by the greater number of PPP proteins upregulated during xylose assimilation, including Xyl2 and Xyl3, which are critical for flux of xylose through the PPP (Fig. 4B). Meanwhile, the almost unchanged abundances in strain CBS7504 of proteins found in the PPP agree with the increased xylitol secretion, decreased cell mass, and lipid degradation phenotypes observed (Fig. 4A). Interestingly, both strains showed similar xylose uptake profiles despite varying cell mass and lipid profiles. This suggests that transporters YALI0C06424g and YALI0F06776g are likely responsible and/or specific for xylose uptake, as indicated by increased protein abundances for both transporters across each strain only during the xylose assimilation phase (Fig. 4D).

In lipid metabolism, YB420 increased proteins involved in TAG biosynthesis while CBS7504 increased proteins involved in β-oxidation and TAG lipase activity, strongly supporting the lipid maintenance and lipid degradation phenotypes of YB420 and CBS7504, respectively (Fig. 5). Accordingly, YB420 increased the abundance of NADPH-generating enzymes which supply critical reducing-equivalents for lipid synthesis and xylose assimilation, including SOL3, ZWF1, ME, and UGA2. Previously, overexpression of SOL3 in Y. lipolytica PO1g increased lipid yield, titer, and content (29). SOL3 does not produce NADPH directly, but instead catalyzes the intermediate reaction of the oxidative PPP that feeds into NADPH-producing enzymes ZWF1 and NADP^+^-dependent 6-phosphogluconate dehydrogenase (29). Additionally, the promoters of ZWF1, ME, and UGA2 exhibited increased expression levels in response to nitrogen limitation, a condition which results in increased lipid accumulation (26). Taken together, the increased protein abundance of NADPH-producing enzymes supports the lipid maintenance phenotype observed by YB420, but future studies are needed to confirm this hypothesis.

Several regulators previously reported to influence lipid accumulation in Y. lipolytica were captured by the proteomic analysis. Of these, YB420 cultures showed increased protein abundances of YAS2, POR1, SNF1, and HSF1. In a previous report, overexpression of YAS2 did not increase lipid accumulation in glucose minimal medium but did significantly increase lipogenesis when acetate was the sole carbon source, suggesting indirect involvements in lipid biosynthesis in Y. lipolytica (29). Overexpression of POR1 in Y. lipolytica resulted in an ∼18% increased lipid content in glycerol medium but showed growth defects in glucose medium (28). Conversely, deletion of SNF1 was reported to increase fatty acid accumulation without the need for nitrogen limitation (11) and overexpression of HSF1 resulted in decreased lipid accumulation with glycerol as the sole carbon source (28). We also identified one regulator (REI1) with increased protein abundance and three regulators (YALI0E30789g, SAK1, and UPC2) with decreased abundance in CBS7504 during lipid degradation. Previously, overexpression of UPC2 decreased lipid accumulation while overexpression of YALI0E30789g and REI1 increased lipid accumulation, which does not support the lipid degradation phenotype of CBS7504 in our study (28). Furthermore, deletion of SAK1 has been shown to result in increased fatty acid content (11) but, in our study, decreased SAK1 protein levels were accompanied by lipid degradation in CBS7504.

While some unique genes between CBS7504 and YB420 were discovered by comparative genomics, our proteomic analysis did not identify any of them associated with the xylose or lipid metabolic phenotypes observed. This evokes the question: what underlying genotypes are causing differences in these phenotypes? While our proteomic analysis suggests regulation plays a significant role in affecting the phenotypes of the strain variants, future investigation should illuminate alterations in genome arrangement, epigenetics, and/or variants in promoter regions that could cause phenotypic divergence between CBS7504 and YB420.

In conclusion, our study highlights that the regulation machinery of pentose and lipid metabolism in Y. lipolytica variants is complex and multifaceted, with many aspects remaining to be discovered and elucidated. Our characterization of Y. lipolytica isolates with phenotypic and genetic diversity, however, sheds light on those proteins supporting lipid accumulation or degradation during fermentation of the nonpreferred biomass sugar xylose, useful for targeted strain engineering for effective conversion of biomass hydrolysates to fuels and chemicals.

## MATERIALS AND METHODS

### Strains.

Y. lipolytica strains CBS7504, YB392, YB419, YB420, YB566, and YB567 were used. CBS7504 is from the CBS-KNAW Culture Collection in Utrecht, The Netherlands. All other strains are from ARS (NRRL) Culture Collection, Peoria, IL. The strains were stored in 20% glycerol at −80°C.

### Medium and culturing conditions.

**Switchgrass hydrolysate preparation.** Liberty switchgrass that had been pelleted and cut with a 4-mm knife mill was used. The biomass was hydrolyzed at 20% solids wt/wt (6). A 20-g dry weight of biomass was added to stainless steel vessels. Then, 80 ml of 0.936% sulfuric acid with 3.72 g/liter Pluronic F-68 was added to each vessel. Eleven vessels per oven run were filled. The 12th vessel was filled with 80 ml of water and contained the thermocouple. The vessels were placed in a Mathis Labomat infrared oven. The following settings were used for the program: temperature = 160°C, heat ramp = 2.6°C, mix settings = 50 rpm, 60 s to the left and 60 s to the right, and cooling temperature = 40°C. Once the vessels had cooled, 4.0 ml of 1.0 M citrate buffer was added to each vessel. Then the pH of the pretreated biomass was adjusted to 4.5 to 5.0 with 30% calcium hydroxide. The vessels were placed back in the Mathis oven for mixing at room temperature. The contents of 11 vessels were transferred to a Fernbach flask with a solid rubber stopper. The following enzymes were added: 29.7 ml Cellic Ctec3 and 5.5 ml Cellic NS-22244. The Fernbach was incubated at 50°C with shaking at 125 rpm for 3 days. After 3 days, the solids were removed using a 0.2-μm filter unit. The liquid fraction was stored at 4°C. Multiple batches were made over the course of a week. The batches were pooled. The liquid was then pH adjusted to 6.0 using 10 N sodium hydroxide. After pH adjustment, the switchgrass hydrolysate (SGH) was filter sterilized and frozen at −20°C until week of use. The SGH was thawed at 4°C overnight. Prior to use, it was amended to a whole hydrolysate with 2.31 g/liter (NH_4_)_2_SO_4_, 1.81g/liter Difco vitamin assay casamino acids, 0.018 g/liter DL-tryptophan, and 0.072 g/liter l-cysteine, and then diluted to 50% vol/vol with water. The diluted and amended hydrolysate is referred to as 50% SGH from here on.

**Culturing conditions.** The yeast stocks were streaked on yeast extract-peptone-dextrose (YPD) agar plates and incubated at 28°C for 24 to 48 h. The plates were stored at 4°C until use. YPD medium, 2 ml in a 16-ml tube, was inoculated by loop for pre-seed cultures. Pre-seed cultures were incubated at 28°C with shaking at 250 rpm for 18 h. Then, 0.5 ml of pre-seed culture was transferred to 10 ml of 50% SGH in 50-ml baffled flasks for seed cultures. Seed cultures were incubated 24 h at 28°C with 250 rpm. The seed cultures were centrifuged to remove supernatant and resuspended in sterile water to A_600_ = 50; 150 ml of 50% SHG was inoculated at an A_600_ = 0.75. DasGip DasBox bioreactors were used for experimental cultures. Each strain was inoculated in triplicate. The following settings were used for the bioreactors: beginning volume = 150 ml; vessel = 250 ml; temperature = 28°C; pH set point = 6.0; agitation = 900 rpm; aeration = 9.0 liters/h; base/acid control: use 2 M HCl and 2 M NaOH for automatic dosing; data collection = dissolved oxygen, temperature, and pH. Cognis Clerol FBA 3107 antifoam was used to control foaming. After inoculation, 200 μl of antifoam was added to each vessel. Antifoam was then added by pipet as needed for the duration of culture growth. Three times a day, a 1.2- to 1.5-ml sample was taken for measuring A_600_, residual sugars, and lipids. A 1.0-ml aliquot was removed from each sample for residual sugars and lipid analysis. The 1.0-ml aliquot was centrifuged to remove the supernatant for residual sugar analysis. The cell pellet was washed twice with deionized water and resuspended up to 1.0 ml with water. The samples were frozen at −20°C until analysis. The remaining sample was diluted for A_600_ measurement. Duplicate samples (2.0 ml) for proteomic analysis were taken several times, including once the OD reached <4.0, 2 to 3 h later, at 44 to 48 h, and a final sample at 68 to 72 h. Samples were kept cold while processing. The samples were centrifuged to remove supernatant, washed with 1.0 ml of chilled water, and then centrifuged again to remove water. The washed cell pellets were then stored at −80°C.

### Analytical methods.

**Lipid quantification.** Lipid analysis was done using a sulfo-phospho-vanillin colorimetric assay as previously reported by Dien et al. (6). For each sample, 1.0 ml of sulfuric acid was added to a glass tube and 50 μl of sample (diluted with water if needed). The tube was heated at 100°C in a dry bath for 10 min. After heating, the tubes were cooled in a room temperature water bath for 10 min. Once cooled, 2.5 ml of the vanillin-phosphoric acid solution was added to each tube. The tubes were mixed and placed in a 37°C incubator for 15 min. They were then cooled in a room temperature water bath, after which the absorbance was measured at 530 nm. The vanillin-phosphoric acid solution (0.12g vanillin, 20 ml water, and 80 ml 85% *O*-phosphoric acid) was made fresh daily for assays. A blank with 50 μl water and four calibration standards were used to create the standard curve. The calibration standards were dilutions of corn oil dissolved in 2:1 (vol/vol) chloroform/methanol and 50 μl of each standard was processed in duplicate along with the samples.

**Metabolites and sugar quantification.** Residual sugars were measured on a Thermo high-performance liquid chromatography (HPLC) system. The system used a Bio-Rad HPX-87H column and a refractive index detector. The column was kept at 65°C with 0.6 ml/min of 5 mM sulfuric acid as a mobile phase.

**Liquid chromatography mass spectrometry (LC-MS) for proteomic analysis**. Y. lipolytica cells harvested at the time points detailed above were resuspended in 100 mM Tris-HCl, 10 mM dithiothreitol, pH 8.0 and subjected to bead beating with 0.5 mm zirconium oxide beads in a Geno/Grinder 2010 (SPEX SamplePrep) for 5 min at high speed (1,750 rpm). Samples were adjusted to 4% SDS and incubated at 95°C for 10 min. Crude lysate was then cleared via centrifugation (21,000 × *g*) and quantified by corrected absorbance (Scopes) at 205 nm (NanoDrop OneC; Thermo Fisher). Samples were then treated with 30 mM iodoacetamide for 20 min at room temperature in the dark. Crude proteins (300 μg) were then processed by protein aggregation capture (PAC) (37). Briefly, 300 μg of magnetic beads (1 micron, SpeedBead Magnetic Carboxylate; GE Healthcare UK) was suspended in each sample and protein aggregation was induced by adjusting the sample to 70% acetonitrile. Aggregated proteins were then washed with 1 ml of neat acetonitrile followed by 70% ethanol, and the aggregated protein pellet was digested with 1:75 (wt/wt) proteomics-grade trypsin (Pierce) in 100 mM Tris-HCl (pH 8.0) overnight at 37°C and again for 4 h the following day. Tryptic peptides released from the beads were then acidified to 0.5% formic acid, filtered through a 10-kDa MWCO spin filter (Vivaspin500; Sartorius), and quantified by NanoDrop OneC.

Peptide samples were analyzed by automated 1D liquid chromatography-tandem mass spectrometry (LC-MS/MS) analysis using a Vanquish UHPLC plumbed directly in-line with a Q Exactive Plus mass spectrometer (Thermo Scientific) outfitted with a trapping column coupled to an in-house pulled nanospray emitter, as previously described (38). The trapping column (100 μm ID) was packed with 10 cm of 5 μm Kinetex C_18_ RP resin (Phenomenex), while the nanospray emitter (75 μm ID) was packed with 15 cm of 1.7 μm Kinetex C_18_ RP resin. For each sample, 3 μg of peptides was loaded, desalted, and separated by ultra-high-performance liquid chromatography (uHPLC) with the following conditions: sample injection followed by 100% solvent A (95% H_2_O, 5% acetonitrile, 0.1% formic acid) chase from 0 to 30 min (load and desalt), linear gradient 0% to 30% solvent B (70% acetonitrile, 30% water, 0.1% formic acid) from 30 to 220 min (separation), and column re-equilibration at 100% solvent A from 220 to 240 min. Eluting peptides were measured and sequenced by data-dependent acquisition on the Q Exactive MS as previously described (39).

### Bioinformatics and data analysis.

**Comparative genomics.** The following genome assemblies of Y. lipolytica strains were downloaded from NCBI as GenBank files: CBS7504/CLIB89/W29 (GCA_001761485.1), CLIB122 (GCA_000002525.1), YB392 (GCA_003367865.1), YB419 (GCA_003367925.1), YB420 (GCA_003367965.1), YB566 (GCA_003367945.1), and YB567 (GCA_003367845.1). These were imported individually into a KBase narrative as genomes and combined into a genome set using the Build GenomeSet v1.0.1 application. A phylogenetic tree was constructed using Insert Set of Genomes Into Species Tree 2.1.10 application with a neighbor public genome count of 20. Orthologue genes and unique genes shared between isolates were identified with the Compute Pangenome v0.0.7 application using the genome set of the 7 isolates as the input. Finally, the Pangenome Circle Plot v1.2.0 application was used to produce a list of singleton genes for each base genome identified from the pangenome.

**Proteomics.** MS/MS spectra were searched against the Y. lipolytica CLIB122 proteome (UniProt; Nov18 build) appended with nonredundant proteins from strain YB-420 and common protein contaminants using the MS Amanda v.2.0 algorithm in Proteome Discoverer v.2.3 (ThermoScientific). Peptide spectrum matches (PSM) were required to be fully tryptic with 2 miscleavages; a static modification of 57.0214 Da on cysteine (carbamidomethylated) and a dynamic modification of 15.9949 Da on methionine (oxidized) residues. Peptide spectrum matches (PSM) were scored and filtered using the Percolator node in Proteome Discoverer and false-discovery rates (FDR) were initially controlled at <1% at both the PSM- and peptide-levels. Peptides were then quantified by chromatographic area-under-the-curve, mapped to their respective proteins, and areas summed to estimate protein-level abundance. Proteins without 3 valid values in a minimum of 1 biological condition were removed and remaining protein abundances were log_2_ transformed. Missing values were imputed to simulate the mass spectrometer’s limit of detection using Perseus v1.6.10.43 (i.e., normal distribution, width of 0.3, down shift of 1.9, and mode changed to total matrix) (40). Significant differences in protein abundance were calculated via *t* test for each sample (S2, S3, and S4) against the control group (S1 sample) for each strain using an FDR of 0.05, 250 permutations, and s0 of 1.

**Bioinformatics.** Pathway proteins were annotated using the KEGG database (41) and from literature sources where cited. Ontology associations and orthologs for regulator proteins were identified using panther database classification system to facilitate high-throughput analysis (42).

### Data availability.

All raw mass spectra for quantification of proteins used in this study have been deposited in the MassIVE and ProteomeXchange data repositories under accession numbers MSV000085941 (MassIVE) and PXD020854 (ProteomeXchange), with data files available at https://doi.org/doi:10.25345/C55T8C.

10.1128/mSystems.00443-21.1 TABLE S1 Singleton genes of YB392, YB419, YB420, YB566, and YB567. Download Table S1, DOCX file, 0.03 MB. Copyright © 2021 Walker et al. 2021 Walker et al. https://creativecommons.org/licenses/by/4.0/ This content is distributed under the terms of the Creative Commons Attribution 4.0 International license.

10.1128/mSystems.00443-21.2 TABLE S2 Uniquely shared genes (i) between the undomesticated strains YB566 and YB567 and (ii) between the undomesticated strains YB392, YB419, and YB420. Download Table S2, DOCX file, 0.02 MB. Copyright © 2021 Walker et al. 2021 Walker et al. https://creativecommons.org/licenses/by/4.0/ This content is distributed under the terms of the Creative Commons Attribution 4.0 International license.

10.1128/mSystems.00443-21.3 TABLE S3 Proteomic analysis of (i) xylose metabolism and transporters; (ii) lipid metabolism, lipid regulators, lipase, and NADPH-generating proteins; and (iii) all gene-specific regulators. Download Table S3, DOCX file, 0.04 MB. Copyright © 2021 Walker et al. 2021 Walker et al. https://creativecommons.org/licenses/by/4.0/ This content is distributed under the terms of the Creative Commons Attribution 4.0 International license.

10.1128/mSystems.00443-21.4 FIG S1 Characterization of CBS7504 growing in switchgrass hydrolysate. (A) Optical density measured at 600 nm. (B) Concentrations of glucose, xylose, acetate, and xylitol. (C) Neutral lipids. (D) pH. (E) Volumes of base and acid added. (F) Percent of dissolved oxygen. Data were collected from triplicate bioreactor runs. Download FIG S1, TIF file, 0.2 MB. Copyright © 2021 Walker et al. 2021 Walker et al. https://creativecommons.org/licenses/by/4.0/ This content is distributed under the terms of the Creative Commons Attribution 4.0 International license.

10.1128/mSystems.00443-21.5 FIG S2 Characterization of the undomesticated strain YB392 growing in switchgrass hydrolysate. (A) Optical density measured at 600 nm. (B) Concentrations of glucose, xylose, acetate, and xylitol. (C) Neutral lipids. (D) pH. (E) Volumes of base and acid added. (F) Percent of dissolved oxygen. Data were collected from triplicate bioreactor runs. Download FIG S2, TIF file, 0.2 MB. Copyright © 2021 Walker et al. 2021 Walker et al. https://creativecommons.org/licenses/by/4.0/ This content is distributed under the terms of the Creative Commons Attribution 4.0 International license.

10.1128/mSystems.00443-21.6 FIG S3 Characterization of the undomesticated strain YB419 growing in switchgrass hydrolysate. (A) Optical density measured at 600 nm. (B) Concentrations of glucose, xylose, acetate, and xylitol. (C) Neutral lipids. (D) pH. (E) Volumes of base and acid added. (F) Percent of dissolved oxygen. Data were collected from triplicate bioreactor runs. Download FIG S3, TIF file, 0.2 MB. Copyright © 2021 Walker et al. 2021 Walker et al. https://creativecommons.org/licenses/by/4.0/ This content is distributed under the terms of the Creative Commons Attribution 4.0 International license.

10.1128/mSystems.00443-21.7 FIG S4 Characterization of the undomesticated strain YB420 growing in switchgrass hydrolysate. (A) Optical density measured at 600 nm. (B) Concentrations of glucose, xylose, acetate, and xylitol. (C) Neutral lipids. (D) pH. (E) Volumes of base and acid added. (F) Percent of dissolved oxygen. Data were collected from triplicate bioreactor runs. Download FIG S4, TIF file, 0.2 MB. Copyright © 2021 Walker et al. 2021 Walker et al. https://creativecommons.org/licenses/by/4.0/ This content is distributed under the terms of the Creative Commons Attribution 4.0 International license.

10.1128/mSystems.00443-21.8 FIG S5 Characterization of the undomesticated strain YB566 growing in switchgrass hydrolysate. (A) Optical density measured at 600 nm. (B) Concentrations of glucose, xylose, acetate, and xylitol. (C) Neutral lipids. (D) pH. (E) Volumes of base and acid added. (F) Percent of dissolved oxygen. Data were collected from triplicate bioreactor runs. Download FIG S5, TIF file, 0.2 MB. Copyright © 2021 Walker et al. 2021 Walker et al. https://creativecommons.org/licenses/by/4.0/ This content is distributed under the terms of the Creative Commons Attribution 4.0 International license.

10.1128/mSystems.00443-21.9 FIG S6 Characterization of the undomesticated strain YB567 growing in switchgrass hydrolysate. (A) Optical density measured at 600 nm. (B) Concentrations of glucose, xylose, acetate, and xylitol. (C) Neutral lipids. (D) pH. (E) Volumes of base and acid added. (F) Percent of dissolved oxygen. Data were collected from triplicate bioreactor runs. Download FIG S6, TIF file, 0.3 MB. Copyright © 2021 Walker et al. 2021 Walker et al. https://creativecommons.org/licenses/by/4.0/ This content is distributed under the terms of the Creative Commons Attribution 4.0 International license.
