# Supplementary material for: Exploring Proteomes of Robust Yarrowia lipolytica Isolates Cultivated in Biomass Hydrolysate Reveals Key Processes Impacting Mixed Sugar Utilization, Lipid Accumulation, and Degradation
Source: mSystems. 2021 Aug 3;6(4):e00443-21. doi: 10.1128/mSystems.00443-21 (PMC8407480; doi:10.1128/mSystems.00443-21)
Supplement: TABLE S1 [file msystems.00443-21-st001.docx]

**Table S1.** Singleton genes of YB392, YB419, YB420, YB566, and YB567.

| **Genome** | **Locus Tag** | **Protein Sequence** |
| --- | --- | --- |
| YB392 | B0I71DRAFT_23206 | MTTQPIFLLLLSLEPPPSQPLSKVKGILLPVANPTIDHSLLHLRDSSNQPTPLFQLPSWLLFQEEKRSSNIERGPRLWLPTGIPGGQEQHQITNILNRFNAILDLVSSSSC |
| YB392 | B0I71DRAFT_165597 | CVSWEELGAVSTVPTVCFLTSVFNSATFCKPLLQQVFVSSAIFVPRLLLLLRPLLWLPLGLLCLPGLLCLPGLLCLLRVLLCFSFLLGRSSSNPPSFFSPTRLVFSAYCTHSCLLQASGQRLPWPSRNT |
| YB392 | B0I71DRAFT_132072 | MVSLIAFLSFSRSASQTFPLNLFSESVLEGEGERLVAILAAIRADFLSHPRPCTFQHRSPFAQIALWKVDPCPCNSDLLGITSTLFNSGDVENTVGINVEGDLN |
| YB392 | B0I71DRAFT_140658 | MTLQTLMVNIRSFQIPLCGVSWGFYLVRVLDIIIESRTDILSYSWGLVTFMFVSTCMWLSYIMIEPHRAREKTRGDVTLVCEVIMTILWLGFLAWVLAEGLVYCSKQSELSHAKPTILDGVYRTSVSHNASQPDYSSTGQIWDLQLVLQFCLWSQPVSYLFLHGL |
| YB392 | B0I71DRAFT_39083 | MGCPEWHFLFGRVAMVPRSQRSLWFIFRVNLLLIFFPQLFGCSWRRVSKFFFFVSPIFQFFPIFQVFFCSV |
| YB419 | B0I72DRAFT_42591 | MLHHCTYLLYVRAYFPVVSCSCSASLLVHHVTSHLGGSLSVPCQFLVSSLSVPCQFLVSFLSAHCQFRFTTLLETCCSRPFYWTSDMLKICIYDTNER |
| YB419 | B0I72DRAFT_173937 | MTFSGKFRQKYADGPFSTSSHQPELIFLIPSPEIAASQPRNGCWRDPIDVCPRDQINATRPSPVLLGCYIATLTIYWDLISYFPDYLAHWFQTYRSMFKGKFP |
| YB419 | B0I72DRAFT_34279 | MLLTKERFSFLLLRLGTGLQPRLVLLLPLEIIPVAVSHEDNHCDHKGPKRGNKDMGTLNQKITQSKPFEYSLLWTELTILSVTWLQNHHSTEQISTTKVLSHEGPLHLLQHPPQDRYLKLF |
| YB419 | B0I72DRAFT_7217 | MFFFRLLVTCIAVSVGLATSTNKSTIYTLKTSTYCTSKCGGQQQGTKATPPTCTDGRESFMEFLMLYIPSSIQQVVRRRPVTTGEWGAEEL |
| YB419 | B0I72DRAFT_133747 | MARKNRQTVLSGEFPLLKSIGLYATTIQGDGNCLFRSLADQLYGKDDLKLAMVIRQSVVDYMKKHSAYFEIFYSSEWDESWEAYIDRMSRSSVYGGNLELAAFASAYQLDVVVYQADLRYVITPIDESKGEQRMELEADQGGERPEKVHIAYHTWEHYSSVRCINGPHCGPPEITSAYLSSYVPKGSDLIADVTKDVGEWKIKQLRESLPFEVPDHLLAKVLKEHEDVGEAVDYFLENGIPEEVEGKEEVVGETKIGRETGNGAPSVTEDVSMKEDTMEQVAEPDATIETQKPTYAKTNIQSPTPTLDKKALRAQKAAERTAKREAKARKEAEDTGTKQKGGKKDKARDKKEKQKARKREKKNEAKASTGGTSVDQSSTAETDYKVVMV |
| YB419 | B0I72DRAFT_148340 | MTEQDDKALKLAKKEAKEAKEAKEAKKEKKEKKEKKEKKEKEKSKKRSAEDDD |
| YB419 | B0I72DRAFT_20302 | MVWGLTAVFASLQRLFCAPSEARRPLWGRRCLHATKSPAKSHISPVQCVPAKLTFDKLASSLALLFPQGWR |
| YB419 | B0I72DRAFT_176868 | MQYDVRGQLKVKYDKCFALNNPPRLNMSTVTISNKYYQTSCYMIRRLNNLSRYGLVFDIDTGMSPYHYRRLSHLRRTGTGRDRRGTLAHRRAMRYFDHSPLMVHSCGGPQEALESLSRLGSAFFLPQMGTSPRSATSKGSKARDENLSSNLSSNWTPYSWNSSLNSTQLHSTPLSINTTSQGIKAFGFDSGTVPVVCFVFQTRFTNTDLQMLTLGRSGTPCLDLDVIW |
| YB419 | B0I72DRAFT_176347 | MGKSHGYRSRTRYMFSRDFKKNGTIPMSVYLKTYKVGDIVDIRANGSIQKGMPHKFYHGRTGIVYNVTKSAVGVIINKVVGNRFMEKRINLRIEHIKHSKCRQEFLDRVKANDLKRKEAKAKNEIVQLRRQPGATREAKTLAIDETNYPLTVAPLAYETFI |
| YB419 | B0I72DRAFT_164308 | MPWGLSRISHQSPEMAPYQDPMVGEYIHQQFVDPNPKVVVYVVDSGVNINHDNFATKPIWLANYADSDDSDANGHGTFVAGVVAGTRSGVDPNLQVKSIKVFSGETTDASILMSGITRAINDFKADTTPGKKAVLNLSLGGDVSTALDSLIKQAVAEGMFVAIAAGNNMENACNNSPGRVSTSTPGSVTVGSIDRSDKLSVYAGNNKGTAWGTCITGFAPGSDIMSSMNTPNDGYGIGPGTSFATPMVAGIAGYLMSQEGTKDLTPAELESRIMNSNDGRIQGDLKNSPNKIAYNGV |
| YB419 | B0I72DRAFT_15024 | MYEYCTCMLLARHFHFTCASLKLVILVHITPWSQWLLYIFPVAIRNVSYLDTVFLVVHLFWRRVTGKISFHLFVVGTINIL |
| YB419 | B0I72DRAFT_2773 | MEGGRIEARKVKLMRRIDSHGCLWILGTCSGLEQLAIPHLWGLESPVLRRLIRELIIEFCVANFGCWRRDIGQQPPRVGSKWSRQHMCRPQSFCSMWDSPISVWFLGHLSLWPGFLFFFFF |
| YB419 | B0I72DRAFT_148966 | MTTSPKPPVNSDAYVNTCHNYGISFNNSAAYHTSSNGLVERRTLILKDMVRSGIAGGCG |
| YB419 | B0I72DRAFT_138964 | MRLACARSPVRFRCRPLFLSLFFVLTKFGRVGFGSLKFVERRANGEARWEIDNHVITFCGMYGKLFDYCSARRMIRHSE |
| YB419 | B0I72DRAFT_134698 | MLNYIKQQIILHLIINPRRSYQFRELRHSTLQPAQPIDIHRTLESYKNVTLLLHLLVYIGSQWLLPCLVSLLILLYLGSFITIFINVSVFLLLTCFAF |
| YB419 | B0I72DRAFT_128204 | MCETTDLGSTNPTIKKLRNASRYRPPAASTFPLLCMQKQITDGYQIGTCQCRVLKQFLLCLKCQLDRHMKKLATGSMTVVAALFVCLMEAFSEVQNEYEYEYEYE |
| YB419 | B0I72DRAFT_141486 | MFSLREKSRDTLPLESTAVDAAASLISTVPQDWSFVKEYNYPGVPPVKTYRREIAGEPWFVRVSEHKDPNDRPYDLFEKFRQGLLLNHTSNEVQYIPMLQGFEQVGHCREYENLVVHYKFPIGLANRKMAVWLLATSLPDEFYIIQFPADNVVEGFKAVYMSVENVRLEGDTVRWTMAQTSDARGFLPRWIQNQSIASAIAQDVQHFVDWVKEKEEEELLLEG |
| YB419 | B0I72DRAFT_6483 | MVKSRATRLLIWSSLLFLNAQMSPRQYHVDVNKQSPEFHGCSRRLLVGIGALEWFLPVYHLNGSLSLSLSLSLSVSLSLSLSGAPGLFVPVFDPPRRVCKWLLVSESGKGCIF |
| YB419 | B0I72DRAFT_26882 | MSSSSSNTYASHVEITSSFAAMLNKNEANDYSSLATEESWIPVAWGEASHKDNAFVSYMGSKKYAEKVAWRFIKSEKPSFGLTTVNPGCVFGSGIALDAKSPNSTTNGTIIQGHLSTTPGQDTSEGQSSCVCHRQNLENQRLMPCVSKLCNQDLRDTVNKEVPDLKGKTMSKPERS |
| YB419 | B0I72DRAFT_159127 | MGERKPVRIHLDCRPLGHVSPMSTPRQAAVICYSSRMHWAGSYAVGTSATDDIVQDGLRGS |
| YB419 | B0I72DRAFT_164433 | MSLFLSSNRLYASVSACVTSVPESLGLSRLCLFRVNSKRSDEKRGLSLNGNCTCTVVRKSPEIEPVIVSRDFKLDIVTYYYKYCTHRTVHRNRHTDPDRISQLHKQKDSDADSDTDSDTDSDTKSDTDSDKIRQNPTKFDKIRHTPETKSEKESDKTPANHSNSIQHATNNHLHTGAHFHHPRDLYCRFGDD |
| YB419 | B0I72DRAFT_172193 | MSRAPPCPCGPHTRCQKLCHILRRLRTHAAPEELLLRNRHPPAGGLAGPNWQVKHPVGTMGNFFHTWSRAAEQRAKSSSRAAQYGDISAIILGGILVVFLNF |
| YB419 | B0I72DRAFT_142595 | MVPGCASAYFLMCLVFLVATVLSRSASLMRARRSSLYLFLTSLALSIIPSTCSPSASSTSFWNSS |
| YB420 | B0I73DRAFT_171833 | MKNTARGQLEMIQGHSSYALRVSISISPTYILVLLLVSLYKYSYTRNSPSSVNVKSLGVLGRCGSTFLRVDSGKIPENVTLGGLHNGPTEEIKFHLMAIKLLGNGAIDKVRSWGCRHGSDKWEADFELRDIKNSAGMFFLTISFLERGSE |
| YB420 | B0I73DRAFT_39464 | MFFCCCATNYRTLSHGASLLCLGTAATCLFSFLCQDSLGFSATPTFFSPLFQLAFFQLASFLANLAPSYSYSTLLCYDLYRTDLSVHPIPSCRVQKQPEMLFNRALRLIGQ |
| YB420 | B0I73DRAFT_141110 | MALKLTVSRLVLVSSISHSISVVLVDGLKSGNVDTGDSEIVVTSALVSEWTLFSLLLSDSLFFQWNLKSHILIKYKTELTIRPSLSYRSCTLAVLRLRFHTVSALAAHYFFPKQMGLS |
| YB420 | B0I73DRAFT_11141 | MFLFYLLNHFVKEIKRMHHDISTQKELGIEGRWGNIWDSSLCRETEGCFFCLFVCLFVCLLCFFFFFFFFFFFNFFFFFFFFFFFFFFFFFFGGFSKTSQFLRYTTLILPVQFGLLYEYNCMFGKEHEWEVVKQILKGGIWSHLFEKR |
| YB420 | B0I73DRAFT_166406 | MTSAWVWVLFSPSELSVWTLVGKGTRHWILQIKSSFLTPSRVVYELFFLLTNVQVSIGLVTRRFLLSSCQADDKAQDSQHQPDTPQIYSIATCQLIFHHHSLADPSHEPS |
| YB420 | B0I73DRAFT_141163 | MRPLFSCSFLSVWLPPVAFDIVVTCLIAVDVIPLLKLMLNADVDACTTVTEARGYVHVIAFVFRPHCCFHGCPCPSALRGVTAESRSSCSLSWMFSTPSILVSDALM |
| YB420 | B0I73DRAFT_129575 | MWGILSSLLLHSISVFSPTQLSFQYAHVMPCNPGFNQGFATETLSGYVACWIWDVNMVPCQLQNSVLVQYNSFLQVTVHRIN |
| YB420 | B0I73DRAFT_168289 | MIIEQTLMDMDPNDDDDDYEDSGDVLKTLFSLTPLMSQTYVHTTQIHDRRTSTTSSQLQVLNSKFPTPSSQLQVPNSKFPTPSSQLQVPNSKFPTPSSQLQVPNSTSKFCEETRYPTATFNIVRHSLPLRSVASKHMIPCRLDAPNRLNNKEKTHGTEQWHSAHAQRTKRPRQQ |
| YB420 | B0I73DRAFT_10053 | MTGSTDILTLLTATVVWSQEMVTRRLQEDHKKITRRSQEDHKKMVKRTLLTWSHFQPGFSTLFSMYCWIHYCVYFCLNRQLTKFSGSVCCKPDILQDINFGENIDLTAIPYPRFPSIAFITNPTVHTPKFTLMSNSYPWKNLGGLDRDYKS |
| YB420 | B0I73DRAFT_20235 | MTCARSASVSKSLLMVSFLACSEGLRMRVFPVTKDRIPSTLSLHFPPLRTVAVYITAATDIVSGATSVTGSIVTWNGVSFLT |
| YB420 | B0I73DRAFT_129528 | MLTPYTVGLLIEPFSTPQAPGGNKSPRSISATRPLHYQPFISAALRNAPARTIAGTSNQSCENATGFNSVHTGSFSNSAVRTTPLNAPSSSATAPMTAITLQFTIPTMPVTPPFPYQSF |
| YB420 | B0I73DRAFT_138424 | MYNAPDTARLEPLLEGLIYKRRIVDFGQFERRQWHKYKCKYKYKYKYNGVLDAFLYIFSLIIFSFYPTQVQLRFLVSSMKRLSCQWSSRSRSMYSSIPNTVLVQVKPAYGKRSDIVGIITRSLVKETREPTLGVM |
| YB420 | B0I73DRAFT_26166 | ALALVLALALRAPALALVPALALALALVPGLWLWLWFRLWLWLWLWIRGLRLWLWFRLWLWLWLWIRGLRLWLWLGRTFSGQLCLHFWRFCCHDDYYGHGSPLLRTPQLIMDLFNEK |
| YB420 | B0I73DRAFT_136916 | MTHQHATTLVSFRFVSPFFAAPFFLVCNLLHVKTVAGIFSVSVSGLDISPRDRRVDLLGTYRTSCTPRAQHRDATLGPGSLSHFYSLAPLF |
| YB420 | B0I73DRAFT_162507 | MRSQRPCCIWSLSAEGATLPVCQSVELWMALLHSVHKTDVTEDNLQVGAIPCTLVPLYPHFSLLLNLITILNTKAHSTPATIRQDKSGNSPSASSPRIISVDDVGASQWVRKTQGFAFSTLRTRQSLEGGRKSVRHLMRCCCDCVSINWTFTTMILTKFNPYCGKRNKTNNANFRFSCARTGNDNEDEPCGYFIYAMKKDNIWTVSQKYNKPRKHNPHIGAGLVNRVQNVVLGKDPSYSKYKTGTTAATFGDEVASLSDAILT |
| YB420 | B0I73DRAFT_141781 | MYAIITGALAHAAGFVAQRNHPSGLLWLVDPVVVAIAHPVLYRNIAVWALGKMTLASYDGPGVYVHRKVSVGVDGGMTEGIRGVATGSKPAGFWAKITSSPLSLGSLERD |
| YB420 | B0I73DRAFT_144199 | MAKAPGFFDKRRATPVSVDGNKKFYLKHDNNNVTEHIRKQQVALFCDTARDALASAMRRESFFLTGMAGTGKSVVLRQIAYALSKDTTVAITSTTGLSAQNIGGETIHKWCGIQRKQTTFTPRNTEATIRTTTCLIIDECPLLRHYHRP |
| YB566 | B0I74DRAFT_176766 | MFLLPSPPVKRPFPLPVLFPFLLVKSLLQHLQILTPLPFPLLARSLPLVPRPVLFLHLVSSRASRLDLPLHSLVTMVLLPLSPSRQKALESTLENWTLPPLRSPLLHLQLVLFPLNLRSHLLLEVVEVLPLKTFLDPLLLLAPLLLLAWFLMLLARQLPQVWLLRIFPALSAHFLLPPTCQAPLSLSLPCLLLHLVAVVLLMVVRTDLALVQALRAPALVQALRAPALVQVLRAPALAPALVQVLRAPALVLAQVQVQVLRAPALVQALRAPALVQVLRAPAL |
| YB566 | B0I74DRAFT_42025 | MFGITMSMLLPMSCSLQNEALVFLLCRLWHLLMCLGSCGTFFSFRSISVPSSSHDTHRCQAGSSHLHHVSVFAFPATFPAHTYICFYVHVIPPHVPVISMYRG |
| YB567 | B0I75DRAFT_2109 | MVSVTTTPVTIPSLLVRLEVPGTSPKVVEGTRVTMTLVCPLPSSIVVVPGTEPVSTTSGFTGVGVKVTTTPVTIPSLLVRLEVPGTSPVAVEGTRVTMTLVCPLPSSTVDVPATGPVSTTSGFAGVTVTTTPVITPRSSGTLRVPGVSPVTVEGTTVRT |
