# Supplementary material for: Exploring Proteomes of Robust Yarrowia lipolytica Isolates Cultivated in Biomass Hydrolysate Reveals Key Processes Impacting Mixed Sugar Utilization, Lipid Accumulation, and Degradation
Source: mSystems. 2021 Aug 3;6(4):e00443-21. doi: 10.1128/mSystems.00443-21 (PMC8407480; doi:10.1128/mSystems.00443-21)
Supplement: TABLE S2 [file msystems.00443-21-st002.docx]

**Table S2.** Uniquely shared genes between the undomesticated strains YB566 and YB567 and between the undomesticated strains YB392, YB419 and YB420.

| **YB567** | **YB566** | | **Protein Sequence** | | |
| --- | --- | --- | --- | --- | --- |
| B0I75DRAFT_157292 | B0I74DRAFT_172414 | | MCSMVCISVIYGICSQSLVGSSPITLSRIVQKQSDTVVDPSGTQLPAHYQTSKSPISITGLEDTMQFVVATVSSLKLELPEQEPEQESEQDDFKSLQSLITR | | |
| B0I75DRAFT_40401 | B0I74DRAFT_40877 | | MTPARRNVSRVSTCDAKSYLACPARPTFPLHSDPHAPNSIKVGLRASHFRIQNTKVFNPCCFDGNCTVKYKAAFAFADGPNQYGWAVENAKPTTSKREPSKHACIIDGEKGPASEREHTLGTQGARAWCMLPSYLHRTCIRSPAPSTNCHATGLAKLRSETVAKLKDALNPCCYLYCMLSYILYDCRAKGRKSFGTALIITGY | | |
| B0I75DRAFT_11481 | B0I74DRAFT_21615 | | MTPDATDGVSDWPHWHLCTRPRPFVHGSTLLSRCSRAPSCASPDSDLHGRLRYKGDDKGILKNAARYILTLGATVRTLLQVVQALQTKYFFYATEQVYLSFHASWVYGVWGFGEIWGDLGDVGGVWSVQKHDLGLFRRLIGGSLAIRFFVWPVAQTRPPPHTYQHTTSSLGWCRLVVHIGKVKSPRLSVRLAVVSAPTLRYIYRPWDPVFA | | |
| B0I75DRAFT_140064 | B0I74DRAFT_140479 | | MSRLHGLLLYQGKLFFLPVIRAIHFDSYIFFSGFDLRLRSLLLSIISSASLTEDKVFCHPKKHLNFFFQNGLCMTNAWTKMRHVW | | |
| B0I75DRAFT_154119 | B0I74DRAFT_152545 | | MTHTDSITAEQPLRTKSDVLASIGNLKQRIEEANVALKAATEGDGVPNEQQQEEMAEVSEFVQIYKRNLEREEENLQKLMRGERVNDDCCLNFFEAGITIVLAVDDEFNLPSSACGTSIVSKLIDIGPPPRRLQKHAFSNVRDLLFLGWFNEKNWVNYHDRTARMAYRLTRIGPTYHLKNSDEANLFTFANQRPKSCDKEVCKFD | | |
| B0I75DRAFT_155102 | B0I74DRAFT_149309 | | MSVPDSVLGGFNGCPWSYREIRHELIISKKLPLNGGKGPQIQSRTPHSREIYPGYS | | |
| B0I75DRAFT_128836 | B0I74DRAFT_174097 | | MWRRKGELFKRCCWGWKREIGGPWGLDTCDQGPQAVFARNLPTKTAENASVDVLVCPRLPSPTRHLWSSLLVHLTGVAYTSLRLRRTVGSTYSTLATSGYDTETVV | | |
| B0I75DRAFT_134495 | B0I74DRAFT_134394 | | MTTTMMMTTIGTPLEACLHEPLPWTLTSPSRCPRPRPLTTTSPPRRHLRNHSLTPTSSRRSCLRASRKRHSWTRPTRPPPSQWLQELPLPSLSPTPSETESRLSTTRLLRLPSQLRLPQSPESSRSARLRLTFPLFHPTRFILLSRQPGIMLGARSERQPMLLLTNPRPTRLPNNKRRHTETPRVMKRPKKRNQRLWPRLKHPECLCETEFDCCKNSRRLRQLELRRLLLLKRSERPEKSSRLRSLPQCNPRPREVLWRELPPASRCDL | | |
| B0I75DRAFT_169517 | B0I74DRAFT_163910 | | HGESTPGEHGEHGEHGESTPGEHGEHGEHGESTPGAPGTPAESAPTQAGTPGQASSAPSAPEQANGGVSLGLPLGGLFLAALPLLI | | |
| B0I75DRAFT_137511 | B0I74DRAFT_138950 | | MYNMQKWLVGDARRGDPGFFGRLRVTLCVIAVVGGCVVRGRSGGCAITVVVDVIGCGRDHVWSYARKHGISLMCWFIMCHGKLRGLSVCVICLTANSLGPLLGLFLLLLLIIGQPVGHRTGHELGCRNGENRNQRGRGEQAGGEVLSGGGSNVHGGGVVCCTGRDVMLKDGVSFIVQNRLSQSAG | | |
| **YB392** | | **YB419** | | **YB420** | **Protein Sequence** |
| B0I71DRAFT_39511 | | B0I72DRAFT_38745 | | B0I73DRAFT_40681 | MSSRMTTRQVCVSAASAILAPAVAQLPETRRLVQRTLETKRALLSASKRPSSRSSIDEEATVSEIVLNLNRLPRSFLNRLLRGFLSPLVPPQPVLLFLVCFTATGLITNKIGKRARLASTSNKRVMPRVGMDSCKQIRILQKIKPSFRGKQVIWPHIRNRRIMDLEIVHRGVDVGVTHPQMTHGNLS |
| B0I71DRAFT_12264 | | B0I72DRAFT_37180 | | B0I73DRAFT_14480 | MPSQQLLQLPVLFPRTIAGVAATKSQYSTSISTSTCSVCLSTSVSVSVCLSVCLNSSPSETVACLTHRSRQTAKLPKPPKSPKRQTTSLARHQKKKPRHVPSRRPRLSPKNLHVTCQTLVCFES |
| B0I71DRAFT_131081 | | B0I72DRAFT_138926 | | B0I73DRAFT_133629 | MSQLLGLHLAMFWQEVGSIAATRESILVDLPNEMNNWHVVSPFLDSWGCSRAFGAVLVWRTTRLEKESCQPPTNQMSALQSGS |
| B0I71DRAFT_9972 | | B0I72DRAFT_12030 | | B0I73DRAFT_6620 | MGANLEAVSVWFVACGSPCGVCVFVSVFAGYIRVVMSSPTGSFLLHSVGRLFPLQSSLTGPRHSCFLSLAGEYQTLFRVHLKLILNPSST |
| B0I71DRAFT_130434 | | B0I72DRAFT_134889 | | B0I73DRAFT_131291 | PSWVDVVRGAFFFVALLPTACSNLYFHFERDRRGVIKQRSRVIDTESKRRRPTLEGRESPPRTLAQSRVRVGALDLTPACSQKKITECAWRGRTLKTMILGAMSWCDCSCVLDPGTECWYQ |
| B0I71DRAFT_129034 | | B0I72DRAFT_135679 | | B0I73DRAFT_132750 | MSDSCATDLTCRLTLMILSPLWLIHPLWLIRPLWLALWWLCHRGFVMVASKGGFRTPTIVYKAVHKVVAVCTVVLGRFCISDRSSCVMSAFTGPSTSTLSTCNQ |
