# Supplementary material for: Exploring Proteomes of Robust Yarrowia lipolytica Isolates Cultivated in Biomass Hydrolysate Reveals Key Processes Impacting Mixed Sugar Utilization, Lipid Accumulation, and Degradation
Source: mSystems. 2021 Aug 3;6(4):e00443-21. doi: 10.1128/mSystems.00443-21 (PMC8407480; doi:10.1128/mSystems.00443-21)
Supplement: TABLE S3 [file msystems.00443-21-st003.docx]

**Table S3.** Proteomic analysis of i) xylose metabolism and transporters, ii) lipid metabolism, lipid regulators, lipase and NADPH generating proteins, and iii) all gene specific regulators.

i) Xylose metabolism and transporters

|  |  |  |  | **Difference against S1 (t-test)** | | | |
| --- | --- | --- | --- | --- | --- | --- | --- |
| **Name** | **Symbol** | **Locus Tag** | **Accession** | **CBS_S3** | **CBS_S4** | **YB420_S3** | **YB420_S4** |
| Ribose-phosphate pyrophosphokinase | PRS1 | YALI0B13552g | Q6CER7 | -1.9671 | -4.0513 |  |  |
| NADP+-dependent glucose-6-phosphate dehydrogenase | ZWF1 | YALI0E22649g | Q6C4Y7 |  |  | 1.1303 | 1.4612 |
| 6-phospho-  gluconolactonase | SOL3 | YALI0E11671g | Q6C682 |  |  | 1.1205 | 1.0834 |
| Ribose 5-phosphate isomerase B | RPIB | YALI0F01628g | Q6C393 |  |  | 3.6477 | 2.0476 |
| Transketolase | TKL | YALI0D02277g | Q6CAJ3 | 6.7655 | 6.9156 | 3.1395 | 3.9834 |
| Xylitol dehydrogenase | Xyl2 | YALI0E12463g | Q6C648 |  |  | 1.7309 | 1.9813 |
| D-arabinitol 2-dehydrogenase | ADH | YALI0F02211g | Q6C367 |  |  | 1.4194 | 1.5731 |
| Xylulokinase | Xyl3 | YALI0F10923g | Q6C246 |  |  | 2.5619 | 3.4454 |
| Ribulo and xylulose kinase |  | YALI0D15114g | Q6C916 | 1.6848 | 1.6915 | 2.4755 | 2.3792 |
| Ribulokinase |  | YALI0E13321g | Q6C615 | 1.3009 | 2.0941 | 2.9823 | 3.5476 |

ii) Lipid metabolism, lipid regulators, lipase and NADPH generating proteins

|  |  |  |  | **Difference against S1 (t-test)** | | | |
| --- | --- | --- | --- | --- | --- | --- | --- |
| **Name/Description** | **Symbol** | **Locus Tag** | **Accession** | **CBS_S3** | **CBS_S4** | **YB420_S3** | **YB420_S4** |
| Lysophospholipid acyltransferase | ALE1 | YALI0F19514g | Q6C138 |  |  | 2.6640 | 2.1208 |
| Phosphatidate phosphatase | PAP | YALI0D27016g | Q6C7L9 |  |  | 2.0638 | 2.3710 |
| Acyl-coenzyme A oxidase | POX6 | YALI0E06567g | Q6C6T0 | 1.7056 | 1.7786 | 1.7737 | 1.9875 |
| Acyl-coenzyme A oxidase 1 | POX1 | YALI0E32835g | O74934 | 3.3638 | 3.7053 | 0.8228 | 2.5377 |
| Lipid phosphate phosphatase 1/ Diacylglycerol pyrophosphate phosphatase 1 | LPP1 | YALI0B14531g | Q6CEM4 |  |  | -3.6199 | -3.1463 |
| Acyl-coenzyme A oxidase 3 | POX3 | YALI0D24750g | O74936 | 1.3715 | 1.1459 |  |  |
| Acyl-coenzyme A oxidase | POX5 | YALI0C23859g | F2Z630 |  |  | 1.9468 | 1.5728 |
| Diacylglycerol acyltransferase | DGA1 | YALI0E32769g | Q6C3R2 | 4.4033 | 3.3990 | 2.7158 | 3.5443 |
| acylglycerol lipase | YJU3 | YALI0C14520g | Q6CBY1 |  |  | -0.2037 | -2.1435 |
| Acyl-coenzyme A oxidase 2 | POX2 | YALI0F10857g | O74935 | 2.7218 | 2.7227 | 1.3433 | 1.7141 |
| Acyl-CoA:sterol acyltransferase | ARE1 | YALI0F06358g | Q6C2N7 | 3.6618 | 3.9737 | 4.3017 | 4.6652 |
| Acyl-coenzyme A oxidase | POX4 | YALI0E27654g | F2Z627 | 1.3130 | 1.5064 |  |  |
| 3-ketoacyl-CoA thiolase | POT1 | YALI0E18568g | Q05493 | 1.1584 | 1.4357 |  |  |
| Glycerol-3-phosphate acyltransferase | SCT1 | YALI0C00209g | Q6CDI7 | 1.5656 | 1.4482 |  |  |
| 2nd step of β-oxidation | MFE1 | YALI0E15378g | F2Z6I5 | 1.5369 | 1.5738 | 1.4204 | 1.5607 |
| Acetyl-CoA Carboxylase | ACC1 | YALI0C11407g | Q6CC91 | -1.4799 | -1.6342 | -1.4448 | -1.7277 |
| Fatty acid synthase 1 | FAS1 | YALI0B15059g | P34229 | -1.2545 | -1.2162 |  |  |
| Malic enzyme | ME | YALI0E18634g | Q6C5F0 |  |  | 2.1108 | 2.2345 |
| TAG lipase |  | YALI0B09361g | F2Z5Z7 | 7.2811 | 8.5246 | 0.3558 | 3.9827 |
| TAG lipase |  | YALI0A20350g | F2Z685 | 2.8796 | 2.0287 |  |  |
| TAG lipase |  | YALI0E17655g | Q6C5J1 | 2.8632 | 2.8156 |  |  |
| TAG lipase |  | YALI0E08492g | Q6C6L5 | 2.8734 | 1.0780 |  |  |
| Mannitol dehydrogenase | MnDH1 | YALI0B16192g | Q6CEE9 | 3.3192 | 2.9949 | 2.4013 | 1.7217 |
| Succinate semialdehyde dehydrogenase | UGA2 | YALI0F26191g | Q6C0B4 |  |  | 1.1263 | 1.2628 |
| 6-Phosphogluconolactonase | SOL3 | YALI0E11671g | Q6C682 |  |  | 1.1205 | 1.0834 |
| NADP+-dependent glucose-6-phosphate dehydrogenase | ZWF1 | YALI0E22649g | Q6C4Y7 |  |  | 1.1303 | 1.4612 |
| HLH transcription factor, upregulates cytochrome p450 genes in response to alkanes with YAS1; some similarity to INO4/INO2 from Saccharomyces cerevisiae | YAS2 | YALI0E32417g | Q6C3S8 |  |  | 2.4527 | 3.2634 |
| YALI1D12628g POR1 Subunit of the SWI/SNF chromatin remodeling complex involved in transcriptional regulation | POR1 | YALI0D12628g | Q6C9A9 |  |  | 0.2550 | 1.3080 |
| Snf1 kinase | SNF1 | YALI0D02101g | Q6CAK0 |  |  | 1.4764 | 2.4990 |
| Snf1 Activating Kinase 1 | SAK1 | YALI0D08822g | Q6C9S1 | -3.0703 | -1.8315 |  |  |
| Cytoplasmic pre-60S factor, putative similar to S. cerevisiae YBR267W REI1 Cytoplasmic pre-60S factor | REI1 | YALI0B08734g | Q6CFB1 | 2.1448 | 1.5102 |  |  |
| Conserved hypothetical protein weakly similar to *Ajellomyces dermatitidis* C2H2 finger domain-containing protein |  | YALI0E30789g | Q6C407 | -1.7935 | -2.2970 |  |  |
| Conserved hypothetical protein some similarities with S. cerevisiae YGL073w HSF1 heat shock transcription factor | HSF1 | YALI0E13948g | Q6C5Z0 |  |  | 1.5507 | 1.5147 |
| Conserved hypothetical protein some similarities with *S. cerevisiae* YDR213W UPC2 Sterol regulatory element binding protein | UPC2 | YALI0B15818g | Q6CEG5 | -2.9466 | -3.9206 |  |  |

iii) All gene specific regulators

|  |  |  |  | **Difference against S1 (t-test)** | | | |
| --- | --- | --- | --- | --- | --- | --- | --- |
| **Name/Description** | **Symbol** | **Locus Tag** | **Accession** | **CBS_S3** | **CBS_S4** | **YB420_S3** | **YB420_S4** |
|  |  | YALI0C01375g | Q6CDD5 | 2.0741 | 1.8984 |  |  |
|  |  | YALI0F11979g | Q6C201 | -2.2409 | -2.4642 |  |  |
|  |  | YALI0D01573g | Q6CAM0 | -2.3883 | -2.6992 |  |  |
|  |  | YALI0F22649g | Q6C0Q3 | 3.8490 | 3.2792 |  |  |
|  |  | YALI0E30789g | Q6C407 | -1.7935 | -2.2970 |  |  |
|  |  | YALI0B13992g | Q6CEP9 | 1.4844 | 2.0437 |  |  |
|  | UPC2 | YALI0B15818g | Q6CEG5 | -2.9466 | -3.9206 |  |  |
|  |  | YALI0B05038g | Q6CFP6 | 2.3378 | 2.3075 |  |  |
|  |  | YALI0A21241g | Q6CG89 | 3.6231 | 2.6485 |  |  |
|  |  | YALI0E31845g | Q6C3V5 | -2.6461 | -1.2458 |  |  |
|  |  | YALI0D22660g | Q6C851 | 1.3435 | 1.1878 |  |  |
|  |  | YALI0E18326g | Q6C5G2 | 4.5513 | 4.3731 |  |  |
|  |  | YALI0D13068g | Q6C988 | 0.2455 | -2.2345 |  |  |
|  |  | YALI0C23727g | Q6CAW9 | 0.3765 | 1.6404 |  |  |
|  |  | YALI0F14443g | Q6C1P9 | 2.5914 | -0.0253 |  |  |
|  |  | YALI0E32373g | Q6C3T0 | 1.9578 | 2.6877 |  |  |
|  |  | YALI0F23111g | Q6C0N7 | -3.7821 | -2.2872 |  |  |
|  |  | YALI0D07018g | Q6CA00 | -1.2318 | -0.7097 |  |  |
| Activator of stress genes | ASG1 | YALI0C22990g | Q6CB01 | 1.8380 | 1.7157 | -2.7409 | -4.3116 |
| Regulatory factor X | RFX1 | YALI0E13596g | Q6C604 | 3.5640 | 2.9681 | -2.1784 | -3.7038 |
|  |  | YALI0D05041g | Q6CA84 |  |  | -1.2011 | -1.8146 |
|  |  | YALI0E31669g | Q6C3W4 |  |  | -0.8823 | -1.4116 |
|  |  | YALI0E12507g | Q6C646 |  |  | -2.0746 | -1.7487 |
|  |  | YALI0E05555g | Q6C6X2 | 2.7552 | 2.3715 | 1.7322 | 1.1438 |
|  |  | YALI0F12705g | Q6C1X2 |  |  | 0.4715 | 1.2402 |
|  |  | YALI0D07744g | Q6C9W7 |  |  | 1.5997 | 1.1281 |
|  |  | YALI0C05060g | Q6CD03 | 4.1011 | 3.7774 | 1.9564 | 2.1334 |
|  |  | YALI0F16511g | Q6C1G3 |  |  | 2.5367 | 3.3878 |
|  | YAS2 | YALI0E32417g | Q6C3S8 |  |  | 2.4527 | 3.2634 |
|  |  | YALI0D20394g | Q6C8E2 | 2.7145 | 2.5047 | 2.9231 | 4.0901 |
| Fork-like head | FLH1 | YALI0B15246g | Q6CEJ1 | -1.7663 | -1.5297 | 1.5825 | 1.9928 |
|  |  | YALI0D24167g | Q6C7Z2 | 1.3323 | 1.2292 | 1.4477 | 1.3627 |
|  | POR1 | YALI0D12628g | Q6C9A9 |  |  | 0.2550 | 1.3080 |
|  |  | YALI0B14773g | Q6CEL3 |  |  | 0.8264 | 1.2630 |
|  | HSF1 | YALI0E13948g | Q6C5Z0 |  |  | 1.5507 | 1.5147 |
|  |  | YALI0F25113g | Q6C0F7 |  |  | 1.3937 | 0.9967 |
|  |  | YALI0D14520g | Q6C937 | 2.9181 | 3.1384 | 1.6467 | 2.7861 |
|  |  | YALI0A16588g | Q6CGS6 |  |  | 1.6760 | 1.7870 |
|  |  | YALI0B10692g | Q6CF32 | 2.9553 | 2.8854 | -1.4653 | 2.2820 |
|  |  | YALI0D12078g | Q6C9D5 |  |  | 2.0065 | 2.7916 |
| Species-specific tRNA processing | STP1 | YALI0E24937g | Q6C4N8 | -1.6309 | -2.2926 | 0.9968 | 1.5880 |
|  | RGT1-related | YALI0C07821g | Q6CCP0 | 6.9691 | 5.9016 | 7.6957 | 7.4795 |
|  |  | YALI0B20944g | Q6CDV3 |  |  | 2.4303 | 2.3395 |
|  |  | YALI0E33297g | Q6C3P0 | 4.2899 | 5.0048 | 4.9291 | 4.7900 |
|  |  | YALI0E01606g | Q6C7D9 |  |  | 2.7160 | 2.7840 |
|  |  | YALI0D20086g | Q6C8F5 | 2.0086 | 1.9057 | 5.5620 | 5.3571 |
