# Supplementary figures and images for: Exploring Proteomes of Robust Yarrowia lipolytica Isolates Cultivated in Biomass Hydrolysate Reveals Key Processes Impacting Mixed Sugar Utilization, Lipid Accumulation, and Degradation
Source: mSystems. 2021 Aug 3;6(4):e00443-21. doi: 10.1128/mSystems.00443-21 (PMC8407480; doi:10.1128/mSystems.00443-21)

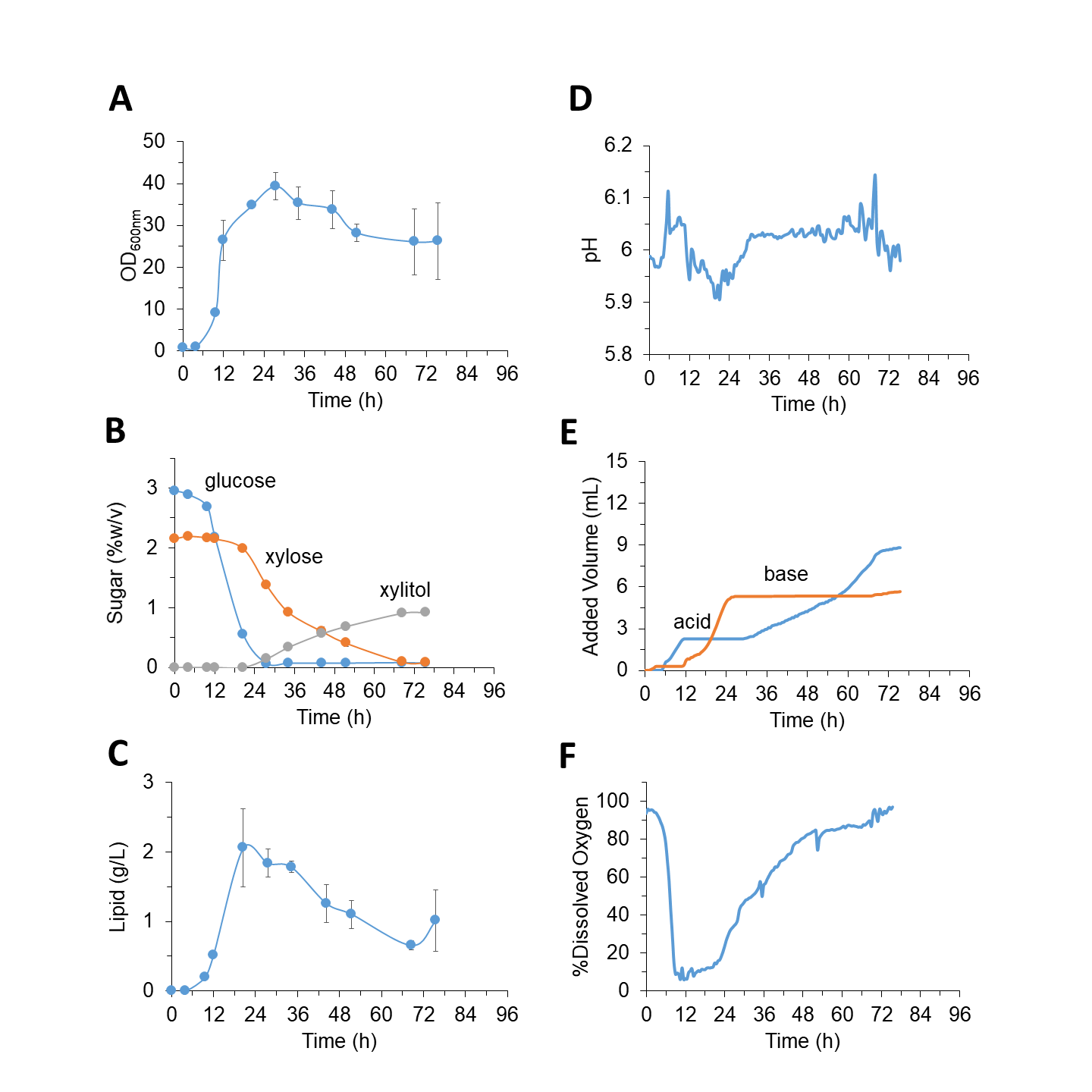

Supplement: FIG S1 [file msystems.00443-21-sf001.tif]

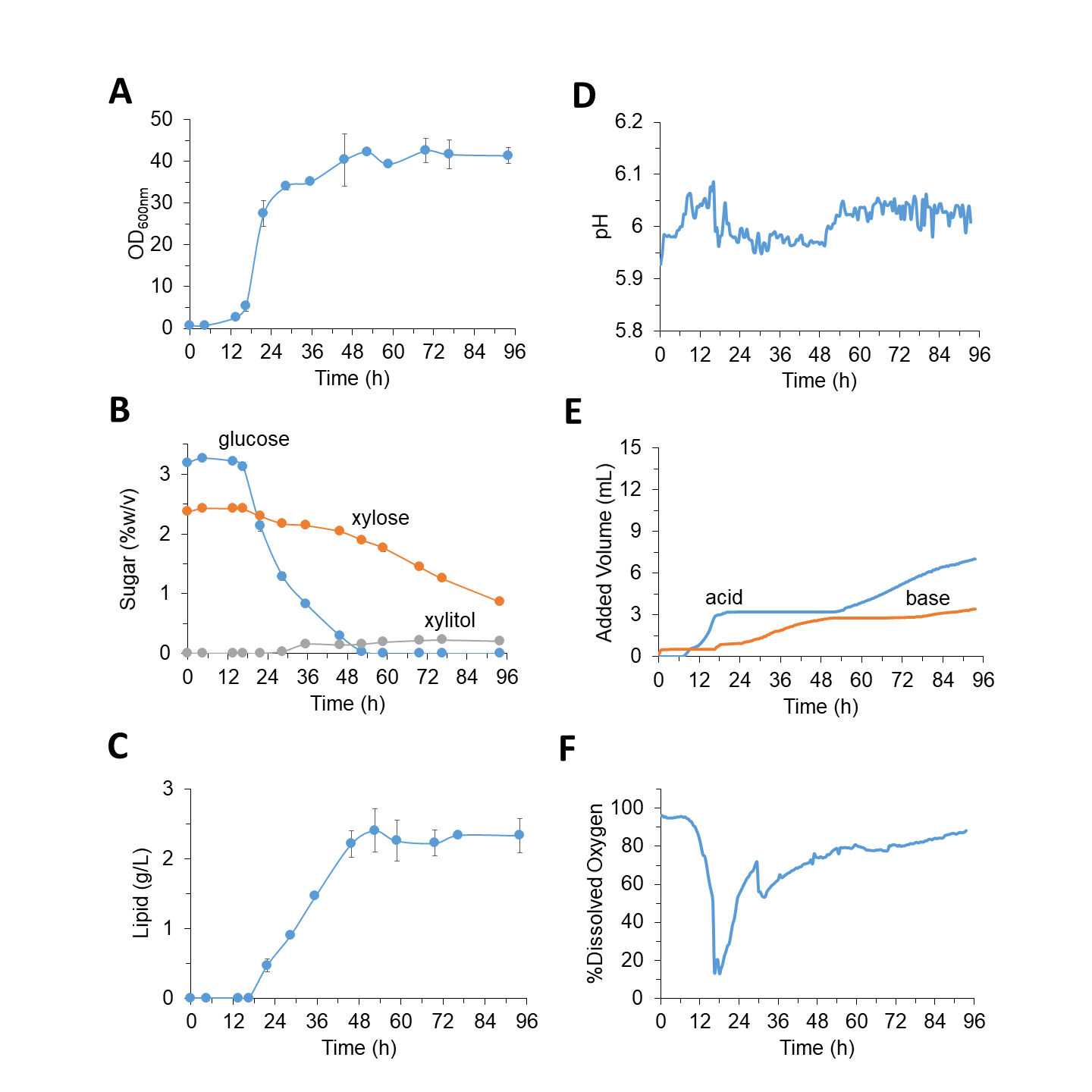

Supplement: FIG S2 [file msystems.00443-21-sf002.tif]

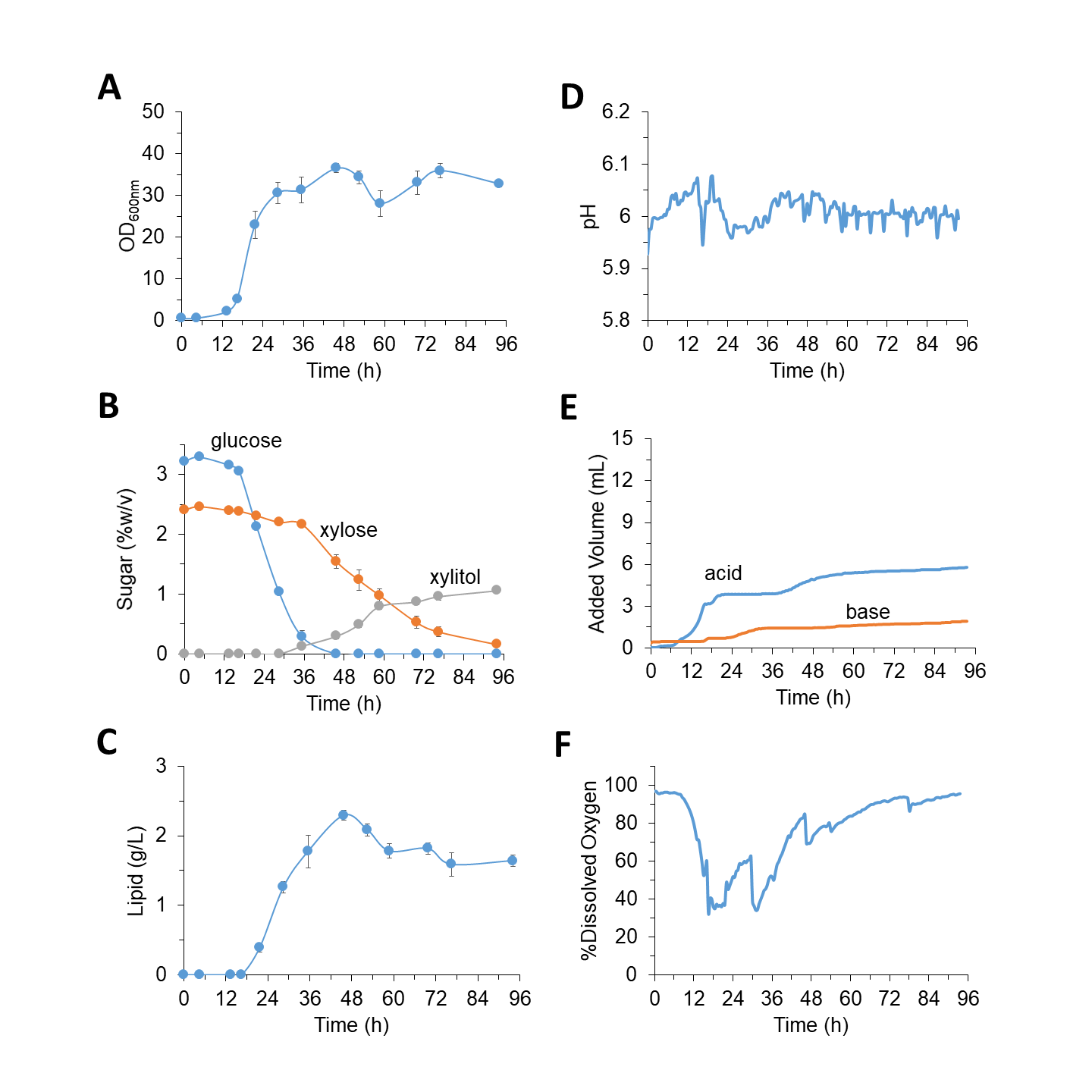

Supplement: FIG S3 [file msystems.00443-21-sf003.tif]

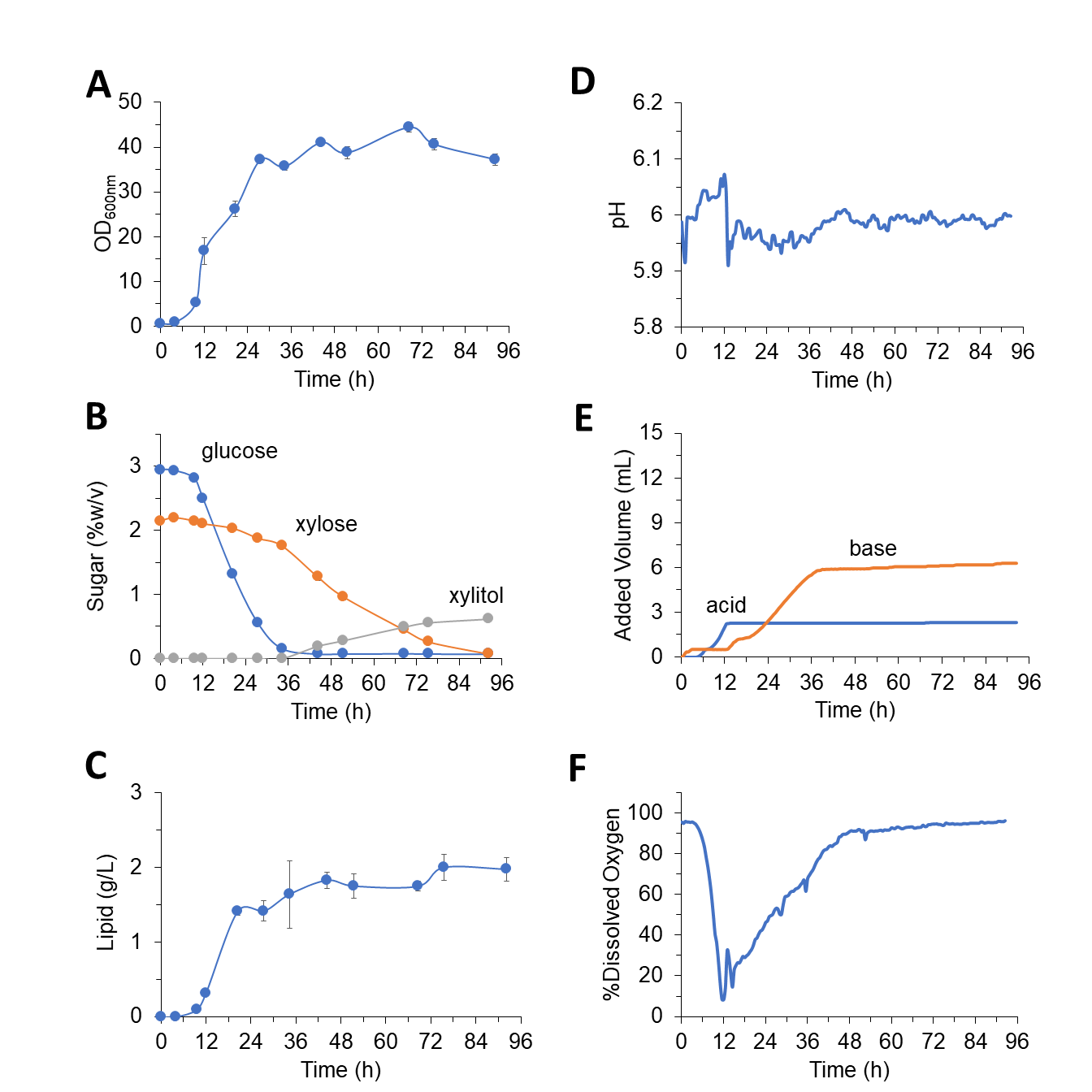

Supplement: FIG S4 [file msystems.00443-21-sf004.tif]

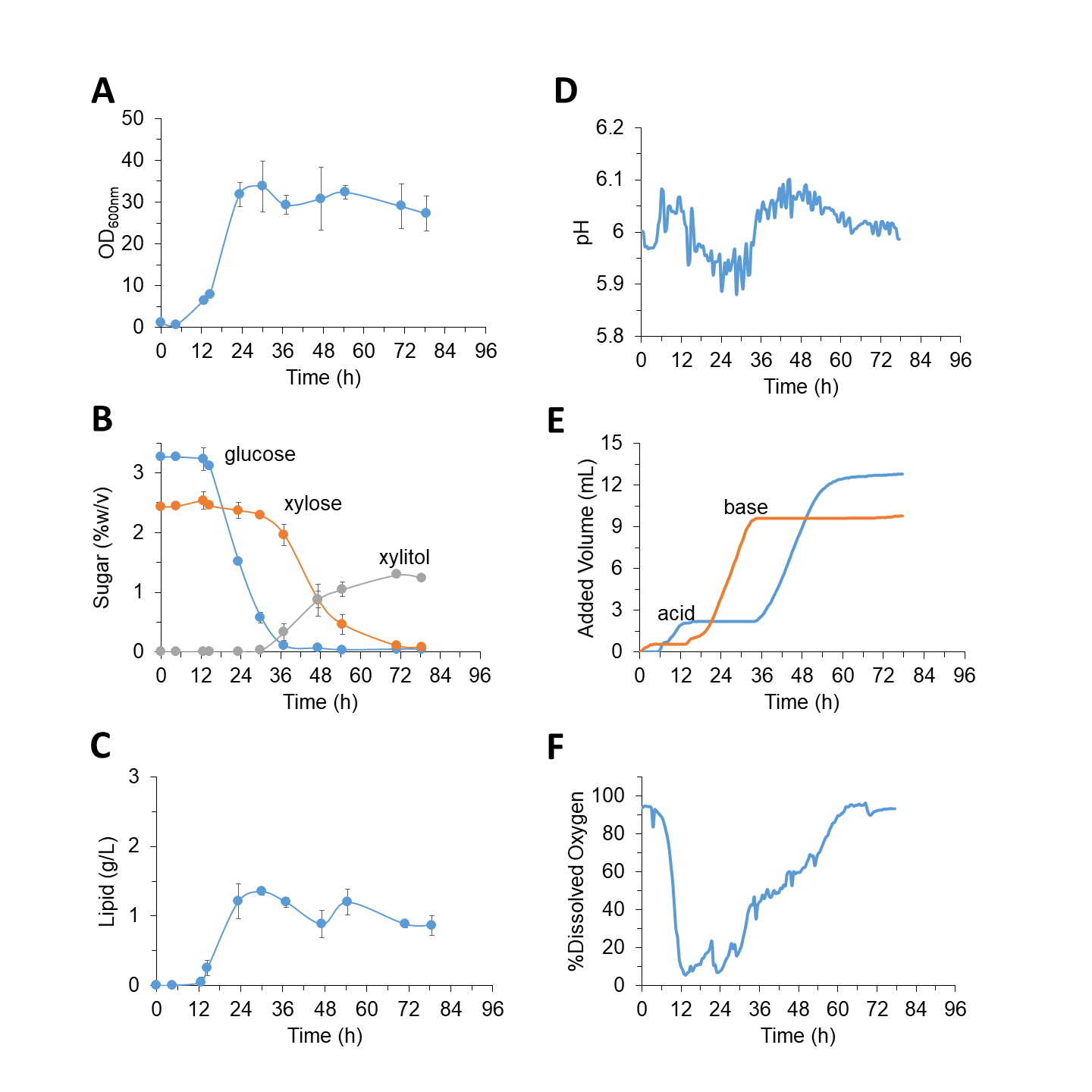

Supplement: FIG S5 [file msystems.00443-21-sf005.tif]

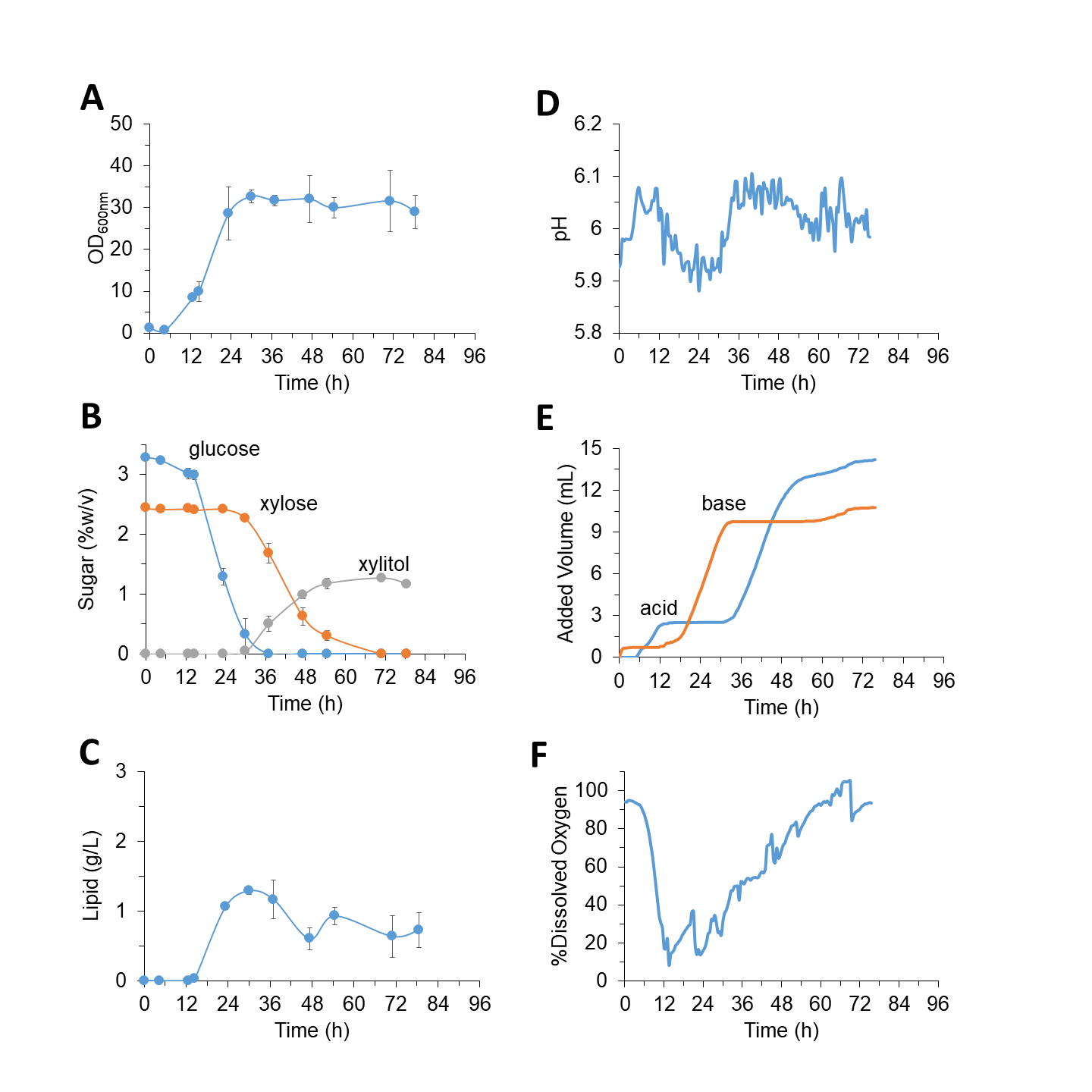

Supplement: FIG S6 [file msystems.00443-21-sf006.tif]
